# Investigating Liquid–Liquid Phase Separation in Lung Adenocarcinoma to Improve Prognostic Accuracy and Treatment Efficacy

**DOI:** 10.1111/jcmm.70807

**Published:** 2025-08-22

**Authors:** Zipei Song, Yuting Li, Pengpeng Zhang, Ke Wei, Miaolin Zhu, Yuheng Wang, Zhihua Li, Liang Chen, Jianan Zheng

**Affiliations:** ^1^ Department of Thoracic Surgery The First Affiliated Hospital of Nanjing Medical University Nanjing China; ^2^ Department of Oncology, the First Medical Center Chinese PLA General Hospital/Medical School of Chinese PLA Beijing China; ^3^ Department of Lung Cancer, Tianjin Lung Cancer Center, National Clinical Research Center for Cancer, Key Laboratory of Cancer Prevention and Therapy, Tianjin's Clinical Research Center for Cancer Tianjin Medical University Cancer Institute and Hospital Tianjin China; ^4^ Department of Oncology The Affiliated Cancer Hospital of Nanjing Medical University and Jiangsu Cancer Hospital and Jiangsu Institute of Cancer Research Nanjing China

**Keywords:** immunotherapy response, liquid–liquid phase separation, lung adenocarcinoma (LUAD), machine learning, prognosis, single‐cell RNA‐seq

## Abstract

Liquid–Liquid Phase Separation (LLPS) refers to the separation of biomacromolecules into separate liquid phases within the cells, plays a critical role in lung cancer pathogenesis. Using machine learning, we developed an LLPS‐associated signature (LLPSAS) based on 79 key genes. The LLPSAS demonstrated superior prognostic performance compared to 140 existing lung adenocarcinoma (LUAD) prognostic models. Patients stratified by LLPSAS risk scores revealed significantly lower overall survival in the high‐risk group. Comparative analysis between the high‐risk and low‐risk groups showed distinct pathway enrichment, genomic alterations, tumour immune microenvironment (TIME) profiles, immunotherapy responses and drug sensitivities. The low‐risk group exhibited an inflamed TIME, suggesting potentially better immunotherapy response. Furthermore, potential effective small molecule drugs were identified for high‐risk patients. Finally, immunohistochemistry confirmed upregulation of LLPS‐associated proteins (PLK1, HMMR, PRC1) in LUAD tissues, and immunofluorescence validated their LLPS occurrence. Conclusively, the LLPSAS provides a valuable tool for LUAD prognosis and treatment optimisation.

## 1 Introduction

Lung cancer represents a prevalent malignancy worldwide and remains a prominent contributor to cancer‐related mortality [1]. Lung adenocarcinoma (LUAD) comprises approximately 40% of all lung cancer cases [2]. For the majority of early‐stage LUAD patients, surgical resection remains the optimal treatment approach, while some advanced‐stage LUAD patients might benefit from chemotherapy combined with targeted therapies [3, 4, 5]. Despite advances in treatment, the prognosis for most advanced‐stage LUAD patients remains unsatisfactory. Therefore, the identification of novel prognostic biomarkers and therapeutic agents for LUAD is of utmost importance.

Liquid–liquid phase separation (LLPS), a physical phenomenon within the cell, refers to the biological process in which proteins and other biomolecules segregate into distinct liquid phases [6, 7, 8]. The multivalent interactions between disordered regions of proteins or RNA molecules are fundamental prerequisites for the occurrence of LLPS within the cell. These interactions drive their aggregation, leading to the formation of liquid droplets, which can transiently organise into ordered structures within the cell known as biomolecular condensates [6, 9]. The formed biomolecular condensates promote the compartmentalisation of ordered structures within the cell, leading to the formation of distinct functional regions. Research on LLPS began in 2009 when researchers discovered P granules in the nematode *C. elegans* . P granules, the liquid‐like structures that consist of proteins and RNA, representing the earliest identified membraneless organelles formed through LLPS [10]. Following that, various substructures, including nucleoli, paraspeckles, Cajal bodies and promyelocytic leukaemia bodies, formed by the occurrences of LLPS within the cell nucleus, along with stress granules located within the cytoplasm, were subsequently identified [11, 12, 13]. The formation of subcellular compartments coordinates the spatiotemporal organisation of various cellular processes. Therefore, LLPS plays crucial biological roles within the cell and is extensively involved in processes such as gene expression regulation, protein degradation, autophagy, metabolic flux regulation, DNA damage and repair and intracellular signal transduction [6, 14, 15, 16, 17, 18, 19]. These processes are key events in tumour initiation and progression.

These years, associations between tumorigenesis and biomolecular condensates formed through LLPS have been increasingly identified. Paraspeckles, a nuclear condensate, have been found to be significantly associated with LLPS. It has been reported that the tumour suppressor gene p53 induces the formation of paraspeckles by upregulating the long non‐coding RNA nuclear‐enriched abundant transcript 1 for response to oncogenic stimuli [20, 21]. LLPS is involved in cellular DNA damage and repair processes, which are closely related to the chemoresistance of cancer cells. Researchers discovered that in colorectal cancer, SUMOylated RNF168 undergoes LLPS, forming nuclear condensates and reduces the efficiency of the DNA damage response (DDR). Furthermore, SENP1, which reduces the SUMOylation of RNF168, weakens this process, thereby enhancing the efficiency of DDR and the resistance of cancer cells to DNA‐damaging agents [22]. With the deepening understanding of the biological mechanisms underlying lung cancer, researchers have discovered that LLPS plays a critical role in lung cancer. Researchers found the LLPS of acetylation‐mediated EZH2 causing the sequestration of its substrate STAT3 within the condensates and leading to the sustained activation of STAT3, thereby promoting carcinogenesis in lung tumour cells [23]. The development of targeted drugs against this process may have potential therapeutic implications for lung cancer. Although improvement in the understanding of LLPS in lung cancer, the mechanisms by which LLPS influences lung cancer initiation and progression remain to be further elucidated. In particular, the association between LLPS and the prognosis as well as treatment response of lung cancer patients has not been sufficiently revealed.

Single‐cell RNA sequencing (scRNA‐seq), a high‐throughput sequencing technique, can be used to study the individual cell level gene expression. Traditional bulk RNA sequencing combines a large number of cells for analysis, which masks the heterogeneity among different cells. In contrast, scRNA‐seq helps to uncover cellular heterogeneity, discover novel cell types and subtypes, investigate cell developmental trajectories, explore cell–cell interactions and signalling pathways, as well as study disease mechanisms [24, 25, 26]. A LLPS‐associated signature (LLPSAS) was developed in LUAD through the analysis of scRNA‐seq data and bulk RNA sequencing data using 101 machine learning (ML) algorithms in this study. Subsequently, the association of the LLPS signature score with the prognosis, tumour immune microenvironment and treatment response was investigated in LUAD patients. The findings of our research introduce a novel prognostic biomarker for LUAD, thereby facilitating treatment decision‐making. Additionally, it provides insights into potential mechanisms of LLPS in LUAD.

## 2 Materials and Methods

### 2.1 Data Acquisition and Preprocessing

The scRNA‐seq data for LUAD, comprising 12 LUAD samples, was obtained from the Gene Expression Omnibus (GEO) database (Accession Number: GSE150938, URL: https://www.ncbi.nlm.nih.gov/geo/). The signature was established by acquiring transcriptome data and clinical data for 1486 LUAD patients from public databases. Samples without clinical information and those with an overall survival (OS) of 0 were excluded from subsequent analysis. The training cohort consisted of 600 LUAD patients from The Cancer Genome Atlas (TCGA) database (URL: https://portal.gdc.cancer.gov/), from which bulk RNA‐seq data, mutation data and clinical characteristics were obtained. To validate the findings, expression profiles and corresponding clinical data of 886 LUAD patients were downloaded from four GEO datasets: GSE30219 (*n* = 85), GSE31210 (*n* = 226), GSE42127 (*n* = 133) and GSE68465 (*n* = 442). Furthermore, RNA‐seq data of 288 normal lung tissues were downloaded from the Genotype‐Tissue Expression (GTEx) database (URL: https://gtexportal.org/home/) for comparative analysis. For the evaluation of immunotherapy response, transcriptome data from the GSE126044 immunotherapy dataset were obtained, comprising 16 NSCLC patients who received anti‐PD‐1 immunotherapy (including five responders and 11 non‐responders). To ensure data comparability, all expression data were normalised to transcripts per million (TPM), except for differential analysis, which utilised counts data format. To account for any confounding effects, the ‘combat’ function from the ‘sva’ R package was utilised to remove potential batch effects. To ensure a more appropriate scale for statistical analysis, a log2 transformation was applied to all data prior to the analysis.

In our study, a total of 3598 LLPS‐related genes were screened from the Data Resource of LLPS (DrLLPS) website (https://llps.biocuckoo.cn/), a comprehensive database established to encompass proteins associated with LLPS (Table S1). For the 3598 LLPS‐related genes, univariate Cox regression analysis was performed using the transcriptome and clinical data from the TCGA‐LUAD dataset. This analysis resulted in the identification of 700 LLPS‐related genes with significant prognostic value (*p* < 0.05). The protein–protein interaction (PPI) network of these LLPS‐related genes was then analysed utilising the STRING website (URL: https://string‐db.org/), filtering out genes that were not connected to the central network. Consequently, a final set of 553 LLPS‐related genes was obtained.

### 2.2 Processing of Single‐Cell Data

In the analysis of the GSE150938 dataset, the scRNA‐seq data from 12 LUAD samples were processed using the Seurat package (version 5.0.1) of the R software (version 4.3.0). Cell quality control was initially conducted to filter out low‐quality genes and cells based on several criteria including the expression of genes in at least 3 cells, a range of 300–7000 genes expressed per cell, mitochondrial gene expression below 10% and red blood cell‐related gene expression below 3%, as well as cells with fewer than 100,000 UMIs. Following these quality control steps, 45,761 high‐quality cells were selected. Subsequently, the data underwent normalisation, scaling and identification of highly variable genes using the ‘NormalizeData’, ‘ScaleData’ and ‘FindVariableFeatures’ functions, respectively. To address any potential batch effects, the ‘RunHarmony’ function was applied. Principal component analysis (PCA) was performed to determine anchor points, and then *t*‐distributed random neighbourhood embedding (t‐SNE) was used for further dimensionality reduction [27]. For the cluster analyses, the ‘FindNeighbors’ and ‘FindClusters’ functions (resolution = 0.5) were applied, resulting in the identification of 16 cell clusters. Marker genes for each cluster were determined by utilising the ‘FindAllMarkers’ function, which compared cells from a particular cluster with cells from all other clusters. This enabled the identification of differentially expressed genes (DEGs) for each cluster, based on thresholds of adjusted *p*‐value < 0.05 and log2 (fold change) > 0.25. Cell types were annotated and identified according to the distinctive marker genes of each cluster. For the analysis of LLPS activity, LLPS activity scores were assigned to individual cells by employing the ‘AUCell’ R package. Following this, cells were classified into high‐ and low‐LLPS‐AUC groups according to the median AUC scores. Visualisation of these groups was performed using the ‘ggplot2’ R package. The ‘cellchat’ R package was employed to evaluate differences in cell communication between the high‐ and low‐LLPS‐AUC groups [28].

### 2.3 Identification of LUAD Molecular Subtypes in TCGA Cohort

In the GTEx database, RNA‐seq data from 288 normal lung tissues were downloaded, aimed to overcome the limited quantity of these samples in the TCGA dataset. Subsequently, this data and the TCGA dataset were integrated into a final dataset comprising 346 normal lung tissue samples and 513 LUAD samples. The data was normalised employing a log2 (count+1) transformation, with batch effects removed. Transcriptomic data for 3598 LLPS‐related genes were obtained from this dataset, among which a total of 191 LLPS‐related DEGs were determined between LUAD and normal tissues (*p* < 0.05, |log2FC| > 1.5). Subsequently, a set of 79 prognostic LLPS‐related DEGs was obtained by performing an intersection between the previously identified 553 prognostic LLPS‐related genes and 191 DEGs (Table S2). To identify LLPS‐based subtypes in LUAD, Non‐negative Matrix Factorisation (NMF) consensus clustering was conducted using the ‘NMF’ R package, based on the expression profiles of the 79 prognostic LLPS‐related DEGs and the TCGA‐LUAD dataset. To evaluate the infiltration of immune cells, seven different algorithms, including TIMER, xCELL, CIBERSORT, CIBERSORT‐ABS, quanTIseq, MCP‐counter and EPIC, were employed [29, 30]. To evaluate the survival differences between the identified subtypes, Kaplan–Meier (K‐M) survival analysis was performed. Additionally, the distribution of immune cells and stromal cells between the two subtypes was assessed using the ‘ESTIMATE’ R package.

### 2.4 Construction of a Prognostic Signature Through 101 Combinations of Machine Learning Algorithms

To create a robust and dependable prognostic signature named as liquid–liquid phase separation‐associated signature (LLPSAS), a combination of 10 machine learning algorithms and 101 algorithm combinations were integrated, including Lasso, Enet, stepwise Cox, plsRcox, SuperPC, RSF, CoxBoost, GBM, Ridge and survival‐SVM. The signature generation process is outlined as follows: (a) A total of 99 prognostic models were established, employing a machine learning computational framework that incorporated 101 algorithm combinations based on 79 prognostic LLPS‐related DEGs. (b) These models were assessed using four validation datasets (GSE30219, GSE31210, GSE42127 and GSE68465). (c) The C‐index for each model was calculated using four validation datasets in order to select the optimal model with the highest mean value of C‐index. (d) Based on the selected model, the genes involved in model development were determined, and risk scores for each sample were calculated. Subsequently, patients were classified into high‐risk and low‐risk groups in each dataset based on the median risk score. This rigorous process ensured the establishment of a reliable and robust prognostic signature, which was validated across multiple datasets.

### 2.5 Model Evaluations and Nomogram Establishment

The ‘survminer’ R package was employed to conduct K‐M curve analysis, investigating potential significant differences in OS and progression‐free survival (PFS) between the two groups characterised by different risk sore levels. To evaluate the prognostic accuracy of LLPSAS, risk, age, gender, as well as stage in predicting OS of LUAD patients, receiver operating characteristic (ROC) analyses were performed applying the ‘timeROC’ package, meanwhile the area under the curve (AUC) of these prognostic factors were calculated and compared. For dimensionality reduction and visualisation of sample distribution disparities between the two groups categorised by different risk sore levels, PCA was employed. Moreover, the correlations between LLPSAS and various clinical characteristics, including age, gender and stage were explored. C‐index values for risk scores and clinical features were calculated and compared using the ‘CompareC’ package across five datasets (TCGA‐LUAD, GSE30219, GSE31210, GSE42127 and GSE68465). Subsequently, a nomogram was constructed by integrating risk scores with clinical variables, aimed to predict the OS of LUAD patients at 1‐, 3‐ and 5‐year. To assess the prognostic accuracy and validate the performance of this nomogram, calibration curves and decision curve analysis (DCA) along with ROC curves were plotted respectively. Finally, a total of 140 published signatures for prognostic prediction of LUAD patients were obtained from a literature search on PubMed. And then the C‐index of LLPSAS and these signatures were compared in the TCGA, GSE30219, GSE31210, GSE42127 and GSE68465 datasets.

### 2.6 Functional Enrichment Analysis

To investigate the potential biological mechanisms related to LLPSAS, Gene Set Variation Analysis (GSVA) and Gene Set Enrichment Analysis (GSEA) were performed based on the MsigDB database (https://www.gsea‐msigdb.org/gsea/msigdb), by the use of R packages including ‘limma’, ‘GSVA’, ‘ClusterProfiler’, ‘org.Hs.eg.db’, as well as ‘GseaVis’. Enrichment analysis of the DEGs between the two groups with different risk score levels was conducted applying the Metascape website (https://metascape.org/), and the results were presented through bar plots and pathway‐based network diagrams [31]. Enrichment scores of each sample were calculated according to enriched pathways, and then t‐SNE dimensionality reduction was applied for results visualisation using ‘Rtsne’ R package.

### 2.7 Comparative Genomic Variant Profiling the Between LLPSAS High‐Risk and Low‐Risk Cohorts

The GISTIC 2.0 analysis (https://gatk.broadinstitute.org) was performed to identify frequently amplified and deleted regions of the genome. Additionally, the tumour mutation burden (TMB) was calculated using the ‘maftools’ R package [32]. The LUAD patients were divided into high‐ and low‐TMB groups based on the median TMB score. Finally, a comparison was made between the prognoses of these two groups by integrating the clinical information from TCGA.

### 2.8 Assessing the Relationship Between LLPSAS and Immunity

The immune score, stromal score and ESTIMATE score for each patient were calculated using the ‘ESTIMATE’ R package. In order to comparing the potential differences in the TIME between the high‐ and low‐risk groups, correlation analysis was performed for assessment of the relationship between these scores, tumour purity and the risk score. Subsequently, the ssGSEA algorithm was utilised to compare the infiltration of immune cells between the high‐risk and low‐risk groups. Furthermore, For the exploration of potential predictive value of LLPSAS in immunotherapy response, comparative analysis of immune checkpoints (ICs) expression was performed. Transcriptome data from the GSE126044 immune therapy dataset, comprising 16 NSCLC patients (including 5 responders and 11 non‐responders) who underwent anti‐PD‐1 immune therapy, were obtained. And then, this dataset was utilised to calculate the LLPSAS risk scores to predict the response to immunotherapy. Finally, immune phenotype scores (IPS) of LUAD patients were obtained from The Cancer Immunome Atlas (TCIA) database (http://tcia.at/home) for the prediction of their potential responsiveness to immune checkpoint blockade (ICB) drugs.

### 2.9 Prediction of Potential Effective Small‐Molecule Drugs and Drug Sensitivity

For the evaluation of drug sensitivity and further identification of potentially effective small molecule drugs for LUAD patients with different risk score levels, a ridge regression model was generated based on the TCGA transcriptome data. The data used for this analysis including drug sensitivity AUC data from the Cancer Therapeutics Response Portal database (CTRP 2.0, https://portals.broadinstitute.org/ctrp.v2.1/) and the PRISM database (https://www.theprismlab.org/), as well as the cell line expression profiles from the Cancer Cell Line Encyclopedia database (CCLE, https://sites.broadinstitute.org/ccle/). By assessing the Spearman correlation between risk scores and AUC scores (CTRP 2.0: Spearman's *r* < −0.4; PRISM: Spearman's *r* < −0.35), a total of 8 CTRP 2.0‐derived compounds and 20 PRISM‐derived compounds were obtained. To explore the interactions between SB‐743921, Ispinesib and PLK1, HMMR and PRC1 proteins, the 2D structures of small molecule ligands were obtained from the PubChem database, and their 3D structures were generated using Chem Office software. Then, the RCSB PDB database (http://www.rcsb.org/) was utilised to screen for crystal structures with high resolution (protein targets) that serve as molecular docking receptors. The protein structures were further processed using PyMOL software, including operations such as dehydration and dephosphorylation. The compounds were energy‐minimised using the Molecular Operating Environment 2019 software, and the target proteins were preprocessed to identify active pockets. Furthermore, molecular docking was conducted using MOE 2019. The binding activities of this interaction were evaluated based on the binding energies, and the results were visualised using PyMOL and Discovery Studio software. The calculation of the half‐maximal inhibitory concentration (IC50) for widely employed anticancer drugs involved the utilisation of The Cancer Drug Sensitivity Genomics of Cancer Cell Lines (GDSC) database (https://www.ancerrxgene.org/) and the R package ‘pRRophetic’ to evaluate drug sensitivity in both the high‐ and low‐risk groups. To estimate the predictive outcomes, a ridge regression analysis was performed, and the 10‐fold cross‐validation was applied for evaluation of predictive accuracy.

### 2.10 Immunohistochemistry and Immunofluorescence Staining

The collection of tissue samples obtained ethical approval obtained from the Medical Ethics Committee of the First Affiliated Hospital of Nanjing Medical University. These samples, including LUAD samples and para‐tumour samples, were collected on the day of the surgery for subsequent immunohistochemical staining and tissue immunofluorescent staining experiments. Immunohistochemistry (IHC) was performed on PFFE tissue sections by incubating with the following primary antibodies against HMMR (Proteintech, 1:200), PLK1 (Proteintech, 1:200) and PRC1 (Proteintech, 1:200) overnight at 4°C. Subsequently, secondary antibodies conjugated with horseradish peroxidase (HRP) (maxim) were applied and incubated for a duration of 30 min at a temperature of 37°C. The sections were then stained with DAB (3,3′‐diaminobenzidine) and counterstained with haematoxylin for visualisation.

Immunofluorescent (IF) staining was performed on 3 μm formalin‐fixed paraffin‐embedded (FFPE) tissue sections from LUAD patients, with initially baked at 85°C for 15 min and followed by dewaxing and rehydration. Subsequently, antigen retrieval was carried out on slides immersed in the sodium citrate buffer (10 mM sodium citrate, 0.05% Tween 20, pH 6.0) under high pressure for 2 min. After cooling to room temperature (RT), slides were washed with TBST/0.5% Tween and incubated with 3% H_2_O_2_ for 15 min, and then blocked with 1% (w/v) BSA Fraction V (ST023, Beyotime) and 10% goat serum (v/v) (B900780, Proteintech) in PBS for 1 h. Slides were incubated with the following primary antibodies against HMMR (Proteintech, 1:200), PLK1 (Proteintech, 1:200) and PRC1 (Proteintech, 1:200) overnight at 4°C, followed by incubation with the secondary antibodies: Alexa Fluor 488‐conjugated affiniPure Goat antiRabbit IgG (H + L) (1:200, 115‐585‐146, Jackson ImmunoResearch) for 1 h, and nuclei‐staining with DAPI (1:200, D9542, Sigma) at RT for 10 min. Images were captured using THUNDER Imaging Systems (Leica Camera AG).

For cell IF staining, SPC‐A‐1 cells were seeded in 6‐well plates containing sterile coverslips and cultured for 24 h. Then, cells were fixed with 4% paraformaldehyde for 15 min, followed by permeabilisation with 0.2% Triton X‐100 in PBS for 5–10 min, and then blocked with donkey serum for 1 h. After staining as described in the method of tissue IF staining, cells were treated with the mounting media containing DAPI. Images were collected at intervals of 3–5 s using a confocal microscope (Leica Camera AG).

### 2.11 Statistical Analysis

The primary processing of data, along with statistical analysis and results visualisation, was performed using R software (version 4.3.0) and Perl software (version 5.30.0). The evaluation of correlation between two continuous variables was performed through Pearson and Spearman correlation analyses. Categorical variables were compared by the use of the Chi‐square test, while the comparison was performed by the use of either the Wilcoxon rank‐sum test or t‐test when involved in continuous variables. All statistical tests were conducted with two‐sided analysis, and a *p*‐value < 0.05 was deemed statistically significant.

## 3 Results

### 3.1 The Characterisation of LLPS‐Related Genes in LUAD Single‐Cell Data

**FIGURE 1:**
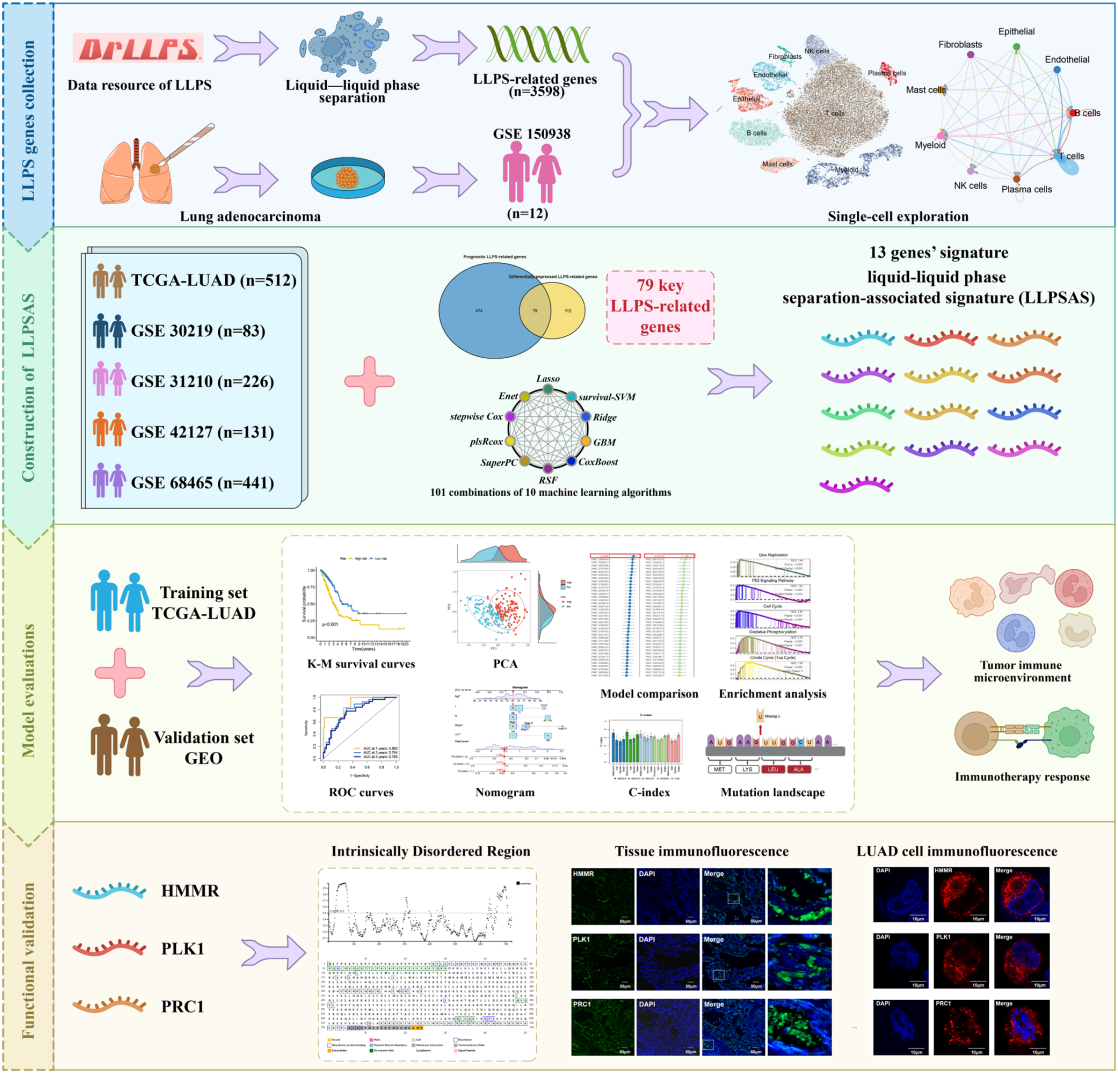
The flowchart of this study.

**FIGURE 2:**
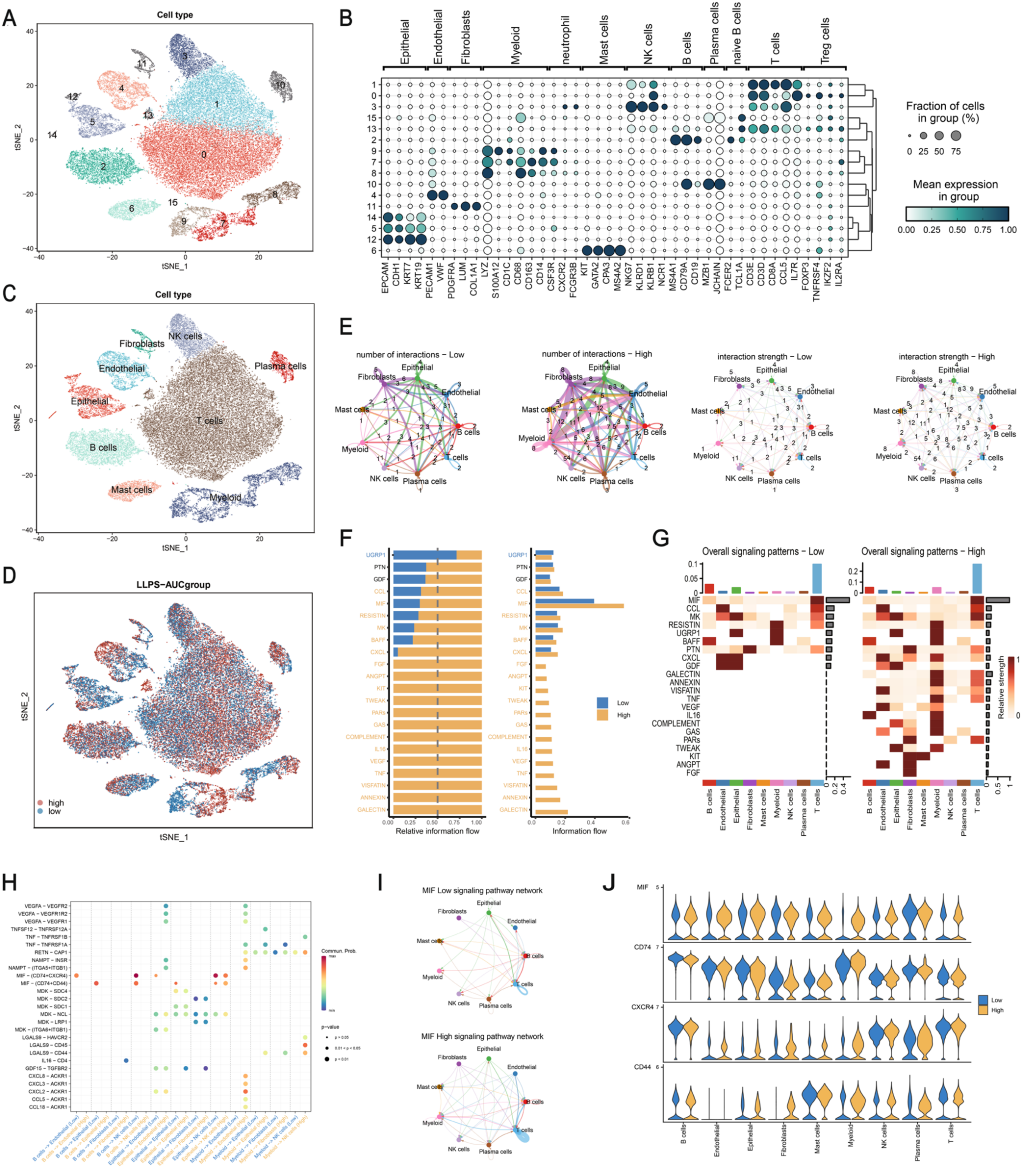
The characterisation of LLPS‐related genes in LUAD single‐cell data. (A) The t‐SNE plot depicted all cells were clustered into 16 clusters following dimensionality reduction and clustering. (B) The association between typical marker genes of diverse cell types in LUAD and the 16 clusters was visually represented through the bubble plot. (C) Cell annotation revealed the presence of nine major cell types. (D) The t‐SNE plot illustrates the distribution of cells from both the high and low LLPS‐AUC groups. (E) The network plot compared the cell communication quantity and cell communication intensity between the high and low LLPS‐AUC groups. (F) Identification and visualisation of signalling pathways in the high and low LLPS‐AUC groups. (G) Comparison of signalling pathway strength between the high and low LLPS‐AUC groups through a heatmap. (H) Identification of upregulated and downregulated signalling ligand‐receptor pairs in different cell subgroups of the high and low LLPS‐AUC groups. (I) Differences in cell communication in the MIF pathway between the high and low LLPS‐AUC groups. (J) Expression patterns of ligand‐receptor pairs in the MIF pathway in different cell populations.

The workflow diagram of this study is illustrated in Figure 1. Firstly, for the screening of 3598 LLPS‐related genes acquired from the Data Resource of LLPS (DrLLPS) website, univariate Cox regression analysis and the STRING website were employed, resulting in the final selection of 553 LLPS‐related genes. The processing of scRNA‐seq data from 12 LUAD patients was beginning with cell quality control, and a total of 45 761 cells of high quality were screened for subsequent analysis (Figure S1A). The expression patterns of the 12 samples were explored, and a significant positive correlation between sequencing depth and total intracellular sequences was observed (*R* = 0.94, Figure S1B). Next, ‘Harmony’ package was used to remove batch effects among samples, resulting in relatively consistent cell distributions across the 12 samples (Figure S1C). Subsequently, dimensionality reduction was performed using PCA and t‐SNE functions, resulting in the classification of all cells into 16 clusters through cluster analysis (Figure 2A). Then, the DEGs and maker genes for each cluster were determined (Figure S1D). The expression distribution of representative marker genes for distinct cell types in LUAD samples and their associations with each cluster were explored for subsequent cell annotation (Figure 2B, Figure S2A). Following annotation, nine major clusters were generated, encompassing T cells, NK cells, B cells, mast cells, epithelial cells, endothelial cells, fibroblasts, myeloid cells and plasma cells (Figure 2C). The differential expression of LUAD‐specific marker genes across these clusters and the distribution differences of cell types among the 12 LUAD samples were also investigated (Figure S2B,C).

The LLPS activity in each cell was evaluated according to the screened 553 LLPS‐related genes, revealing higher expression of LLPS‐related genes in B cells and plasma cells, while lower expression in epithelial cells (Figure S2D). Subsequently, all cells were then categorised into high and low LLPS‐AUC groups according to their median AUC scores (Figure 2D). Differences in cell communication between the two groups were assessed, and the results demonstrated the higher intensities of cell communication in the high LLPS‐AUC group compared to the low LLPS‐AUC group (Figure 2E, Figure S2E). We found that the high LLPS‐AUC group exhibited a general enhancement in the strength of both incoming and outgoing interactions (Figure S2F). Additionally, the higher expression levels of most signalling pathways were observed in the high LLPS‐AUC group (Figure 2F,G). The bubble plot reveals that cells in the high LLPS‐AUC group exhibit enhanced intercellular communication through a greater number of signalling ligands. Particularly, in the interactions between myeloid cells and endothelial cells, ligand pairs such as CXCL3‐ACKR1 and CXCL2‐ACKR1 are upregulated (Figure 2H). Macrophage Migration Inhibitory Factor (MIF) appears to play a crucial role in both the high and low LLPS‐AUC groups (Figure 2F,G and Figure S2G). MIF is well known for its involvement in various biological processes including inflammation, immune regulation, cell proliferation and survival, with potential to be associated with tumour immune evasion and tumour progression [33, 34]. Significant upregulation of the MIF pathway and MIF‐related genes, including CD74, CXCR4 and CD44, was observed in the high LLPS‐AUC group (Figure 2I,J). This finding suggested the potential of the MIF (CD74 + CXCR4) axis to serve as a key signalling pathway in promoting the interactions between different cell populations within the high LLPS‐AUC group. In addition, a significant upregulation of the Chemokine (C‐C motif) Ligand family (CCL) and the Megakaryocyte (MK) pathway was observed in the high LLPS‐AUC group (Figure S2H,I).

### 3.2 Identification of LUAD Molecular Subtypes Based on LLPS‐Related Genes

**FIGURE 3:**
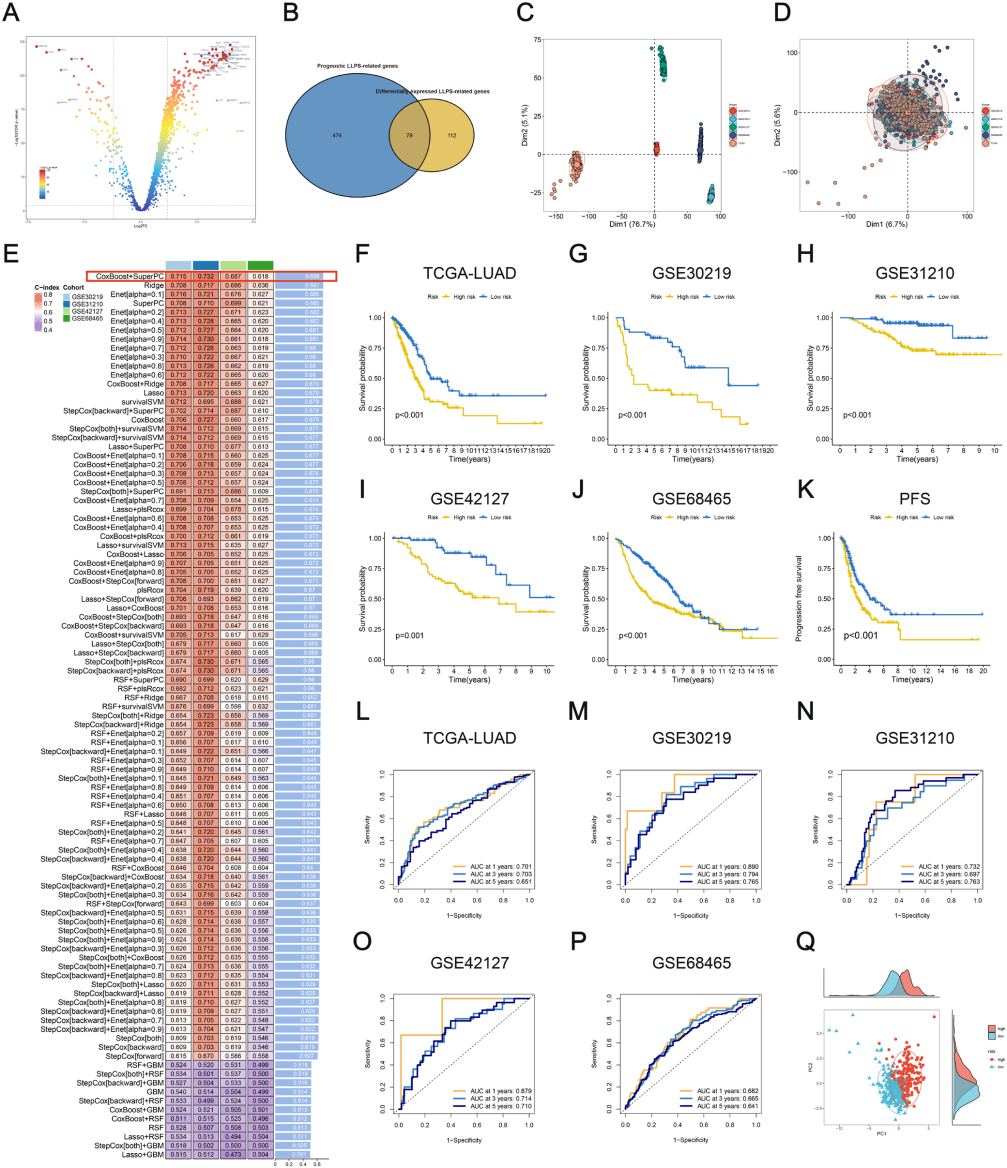
Construction of a prognosis signature based on integrative machine learning. (A) A total of 191 DEGs (*p* < 0.05, |log2FC| > 1.5) were determined and visualised in the volcano plot. (B) The intersection between the 191 DEGs and 553 prognostic LLPS‐related genes was represented in the Venn diagram, resulting in the identification of 79 LLPS‐related DEGs associated with prognosis. (C, D) The distribution of samples in five datasets was demonstrated through PCA plots, both before (C) and after (D) batch correction. (E) Utilising a machine learning computational framework, a total of 99 prediction models were established, and the C‐index of each model was calculated among all validation datasets. (F‐J) K‐M curves of the OS were generated based on the LLPSAS in TCGA‐LUAD (*p* < 0.001) (F), GSE30219 (*p* < 0.001) (G), GSE31210 (*p* < 0.001) (H), GSE42127 (*p* = 0.001) (I) and GSE68465 (*p* < 0.001) (J). (K) The LLPSAS was further assessed using K‐M curves of PFS in TCGA‐LUAD (*p* < 0.001). (L‐P) Time‐dependent ROC curves based on the LLPSAS were plotted for TCGA‐LUAD (L), GSE30219 (M), GSE31210 (N), GSE42127 (O) and GSE68465 (P). (Q) The distribution of patients from the high‐and low‐risk groups in the TCGA‐LUAD dataset was visualised in the PCA plot.

Transcriptomic data of 3598 LLPS‐related genes were obtained from the TCGA and GTEx databases, and batch effects were removed (Figure S3A,B). Subsequent differential analysis identified 191 LLPS‐related DEGs (*p* < 0.05, |log2FC| > 1.5), with 165 upregulated and 56 downregulated in LUAD (Figure 3A, Figure S3C). Then, the intersection of these 191 DEGs with the previously selected 553 prognostic LLPS‐related genes yielded 79 LLPS‐related DEGs associated with prognosis (Figure 3B). Utilising the transcriptomic data of these 79 genes, we conducted Non‐negative Matrix Factorisation (NMF) to identify LLPS‐based LUAD subtypes within the TCGA cohort. According to the status of cophenetic, dispersion and silhouette, the optimal number of clusters was determined as 2 (Figure S3D,E). As a result, a total of 571 LUAD patients were categorised into two subtypes, namely C1 (*n* = 218) and C2 (*n* = 289). Then, the infiltration of immune cells among the two subtypes was assessed by the use of seven distinct algorithms, including TIMER, CIBERSORT, CIBERSORT‐ABS, QUANTISEQ, MCPCOUNTER, XCELL and EPIC. Our findings demonstrated the tumour microenvironment (TME) of C1 exhibited a higher level of immune cell infiltration (Figure S4A). This suggested the TME of C1 attracts a plethora of immune cells, thereby promoting the development of an inflammatory tumour microenvironment. Survival analysis unveiled significantly improved OS and PFS in C1 when compared to C2 (Figure S4B,C). Examination of TME scores for the two subtypes revealed that C1 exhibited a higher immune score, stromal score and ESTIMATE score, while its tumour purity was lower than that of C2 (Figure S4D).

### 3.3 Construction of a Prognosis Signature Based on Integrative Machine Learning

Batch effects were successfully eliminated from the five LUAD datasets (TCGA‐LUAD, GSE30219, GSE31210, GSE42127 and GSE68465) utilised for the construction of our prognostic model (Figure 3C,D). The training set comprised TCGA‐LUAD, while GSE30219, GSE31210, GSE42127 and GSE68465 served as validation sets. For the development of a consensus LLPS‐associated signature (LLPSAS), a total of 101 machine learning algorithms were combined and utilised in the analyses of the previously identified 79 prognostic LLPS‐related DEGs. As a result, a total of 99 models were constructed using different machine learning algorithms (two algorithm combinations did not meet the modelling criteria). Subsequently, we computed the C‐index of each model among all validation datasets (Figure 3E). Notably, the best‐performing model, which combined CoxBoost and SuperPC, exhibited the highest averaged C‐index (0.688). Then, utilising the CoxBoost algorithm, 13 key genes were identified, including CPS1, FGF2, HMMR, KLF4, KRT8, LDHA, MAPK4, MRM1, NME4, PKP3, PLK1, PRC1 and SFN. Subsequently, the risk score for each sample was computed through the SuperPC algorithm utilising these 13 genes, and all patients were grouped into two subsets based on the median risk score.

### 3.4 Model Evaluations and Nomogram Establishment

**FIGURE 4:**
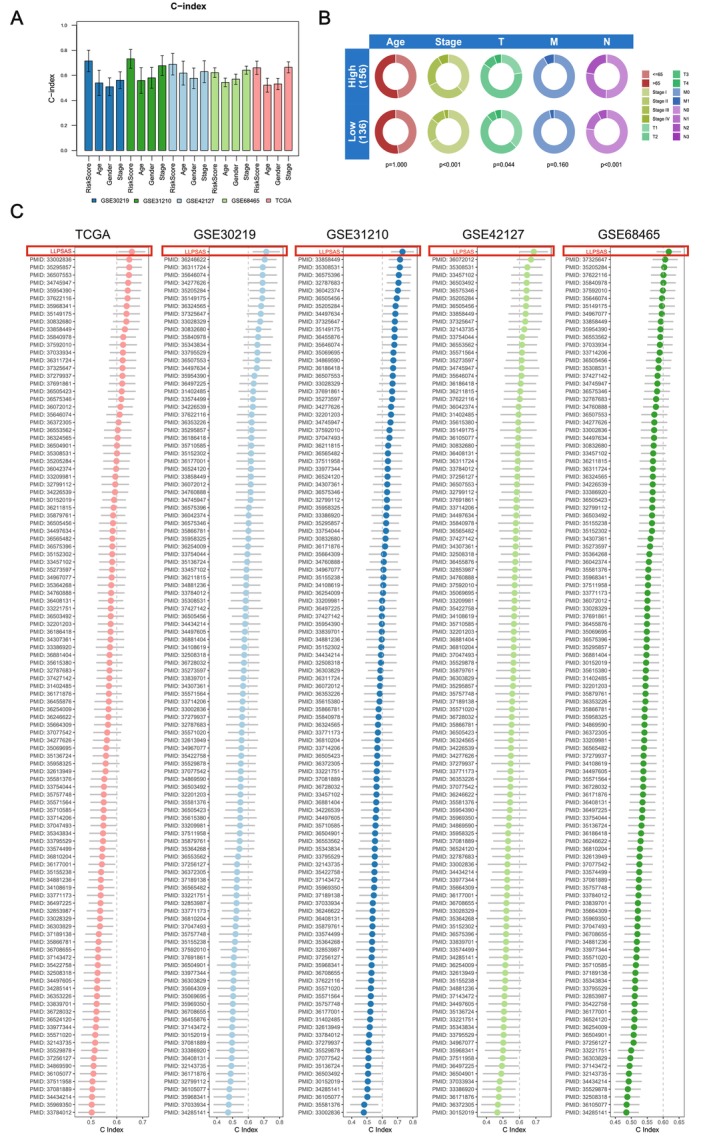
Prognostic performance of the LLPSAS. (A) The C‐index of the risk score and clinical features, such as age, gender and stage, in the TCGA, GSE30219, GSE31210, GSE42127 and GSE68465 datasets, is visually presented in the bar graph. (B) The clinical correlation circle plot displayed the correlation of risk score and age, stage, T‐stage, N‐stage, M‐stage in the TCGA‐LUAD dataset. (C) The C‐index of LLPSAS and 140 previously published LUAD prognostic models in the TCGA, GSE30219, GSE31210, GSE42127 and GSE68465 datasets was calculated and compared.

Among the two groups, the distribution of risk scores, patient survival time and patient survival were further analysed, and the expression pattern of selected 13 modelling genes was compared. The results indicated that in the five datasets, patients from the high‐risk group were more likely with high‐risk scores, shorter survival time and death. Furthermore, the expression levels of CPS1, HMMR, KRT8, LDHA, PKP3, PLK1, PRC1 and SFN displayed a notably positive correlation with the risk scores, which suggested their potential as prognostic risk factors (Figure S5). Subsequent survival analysis was performed separately for the high‐risk and low‐risk groups across the five datasets, and K‐M survival curves were generated. The findings revealed significantly lower OS in the high‐risk group when compared to the low‐risk group across all five datasets (*p* ≤ 0.001, Figure 3F–J). Meanwhile, in the training set, the patients with high risk also displayed poorer PFS (*p* < 0.001, Figure 3K). To evaluate the predictive performance of LLPSAS in LUAD, time‐dependent ROC analysis was performed along with the AUC calculated in each dataset. The AUC values for each dataset at 1‐year, 3‐year and 5‐year were as follows: 0.701, 0.703, 0.651 (TCGA‐LUAD); 0.890, 0.794, 0.765 (GSE30219); 0.732, 0.697, 0.763 (GSE31210); 0.879, 0.714, 0.710 (GSE42127); 0.682, 0.665, 0.641 (GSE68465) (Figure 3L–P). These results demonstrated the potent predictive performance of LLPSAS in prognosticating LUAD patients. Subsequently, for dimensionality reduction and visualisation, PCA was performed to further observe the differences in sample distribution between the two groups. Our results consistently revealed significant differences in sample distribution between the two groups (Figure 3Q, Figure S6A). Additionally, in the ROC curves combined with clinical characteristics, the AUC values for risk and clinical factors such as age, gender and stage were as follows: 0.703, 0.533, 0.517, 0.680 (TCGA‐LUAD); 0.794, 0.523, 0.525, 0.590 (GSE30219); 0.697, 0.634, 0.602, 0.742 (GSE31210); 0.714, 0.682, 0.565, 0.631 (GSE42127); 0.665, 0.550, 0.576, 0.676 (GSE68465) (Figure S6B). LLPSAS demonstrated superior predictive performance compared to other clinical features in the TCGA‐LUAD, GSE30219 and GSE42127 datasets. Furthermore, the C‐index values for risk score were calculated and compared with age, gender, stage and other clinical features. As a result, in the GSE30219, GSE31210 and GSE42127 validation datasets, the risk score exhibited the highest C‐index (Figure 4A). Moreover, our findings revealed a positive correlation of risk score with T‐, N‐ and clinical stage in the TCGA‐LUAD dataset (*p* < 0.05) (Figure 4B).

Considering the universal application of TNM‐staging along with the potent predictive performance of LLPSAS, we developed a nomogram through a combination of TNM‐staging and LLPSAS in the training set to enhance the prediction of the 1‐, 3‐ and 5‐year prognosis. Then, the score of each patient was calculated using the nomogram, enabling a more accurate prognosis assessment (Figure S7A). Additionally, Cox analysis was conducted based on this nomogram; as a result, the highlighting age, stage and risk score were determined as the critical prognostic factors (*p* < 0.05, Figure S7B). Furthermore, we assessed the predictive accuracy of this nomogram across both the training and testing sets. Since the GSE31210 and GSE42127 datasets did not include clinical information related to TNM staging, we selected the GSE30219 and GSE68465 datasets as the testing set for validation. Consistency between the predicted and observed values was demonstrated by calibration curves (Figure S7C–E). Additionally, time‐dependent ROC analysis was performed utilising this nomogram in the training set (TCGA‐LUAD) and the testing set (GSE30219 and GSE68465). The AUC values for 1‐year, 3‐year and 5‐year were as follows: 0.756, 0.737, 0.728 (TCGA‐LUAD); 0.714, 0.785, 0.753 (GSE30219); 0.772, 0.782, 0.749 (GSE68465) (Figure S7C–E). The usefulness and effectiveness of this nomogram were assessed using DCA, revealing its potential in accurate prediction for the survival probability of LUAD patients at distinct time intervals (Figure S7F). The results demonstrated that our nomogram has a robust prognostic predictive performance in LUAD patients.

### 3.5 Comparison of LLPSAS and Previous Signatures in Predicting the Prognosis of LUAD

In the past decades, the significant advancement in next‐generation sequencing and bioinformatics technologies accelerates the development of numerous machine learning‐based signatures for the prognostic prediction of LUAD patients. With the aim to assess the predictive capability of LLPSAS in greater detail, we conducted a comprehensive search of PubMed and retrieved 140 published signatures, including mRNA and non‐coding RNA signatures, associated with diverse biological processes such as cellular immunity, ageing, metabolism, autophagy, ferroptosis, cuproptosis, necroptosis, m6A RNA methylation and inflammation. Subsequently, we performed a comparison for the predictive performance of LLPSAS and other models in the TCGA, GSE30219, GSE31210, GSE42127 and GSE68465 datasets, respectively, by examining the C‐index (Figure 4C). The findings consistently revealed that LLPSAS outperformed all other models in each dataset.

### 3.6 Underlying Biological Mechanisms Associated With LLPSAS

**FIGURE 5:**
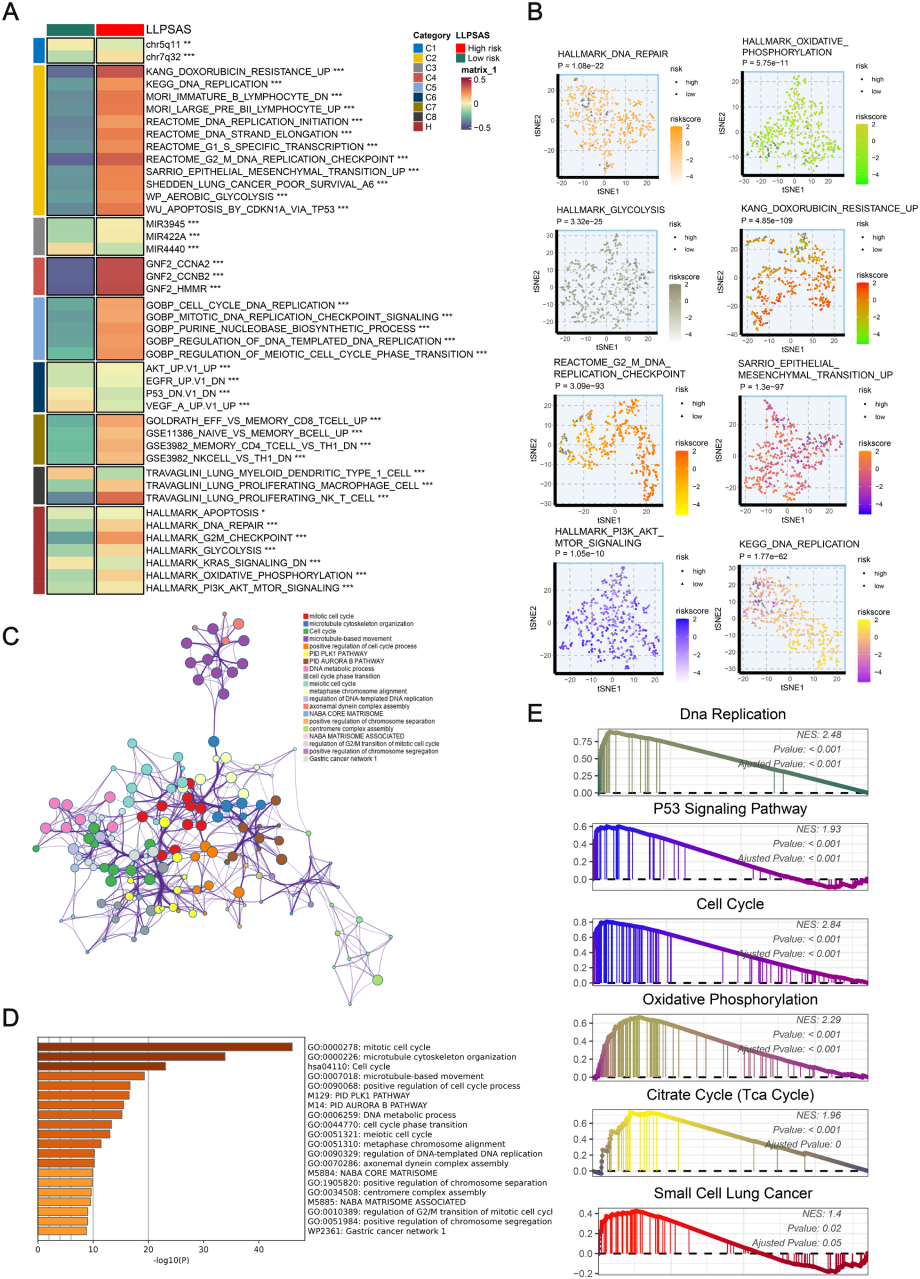
Underlying biological mechanisms associated with LLPSAS. (A) The biological features of the high‐ and low‐risk groups were depicted through GSVA analysis. (B) Differences in pathway activity between the two groups were illustrated in the t‐SNE plot. (C, D) Metascape was utilised to perform enrichment analysis on DEGs between the two groups, and the results were visualised through the network plot (C) and bar graph (D). (E) GO and KEGG terms related to LLPSAS were subjected to GSEA analysis.

LLPS is widely involved in gene expression regulation, DNA damage and repair, autophagy, metabolic regulation and signal transduction in cells, which are critical events in tumour initiation and progression [6]. Therefore, we attempted to uncover the potential biological mechanisms associated with LLPSAS and its correlation with the tumour biology processes in LUAD. The results of GSVA analysis revealed that the risk score of LLPSAS exhibited a significantly positive correlation with various pro‐oncogenic pathways, including DNA replication, cell cycle regulation, epithelial‐mesenchymal transition (EMT), glycolysis, oxidative phosphorylation and PI3K‐AKT–mTOR signalling (Figure 5A). Furthermore, we found that samples could be distinguished based on the risk scores of LLPSAS, calculated as enrichment scores for the pathways in each sample and reduced to a two‐dimensional plane (Figure 5B). The Metascape analysis demonstrated that the DEGs between the two risk groups were enriched in pathways associated with cell cycle regulation, DNA metabolism and DNA replication (Figure 5C,D). Additionally, the findings of GO, KEGG and GSEA analyses revealed the enrichment in pathways related to DNA replication, cell cycle, oxidative phosphorylation, p53 signalling pathway, tricarboxylic acid (TCA) cycle and small cell lung cancer in the high‐risk group, which is consistent with our previous findings (Figure 5E).

### 3.7 The Genomic Variation Landscape Between High‐ and Low‐Risk Groups of LLPSAS

**FIGURE 6:**
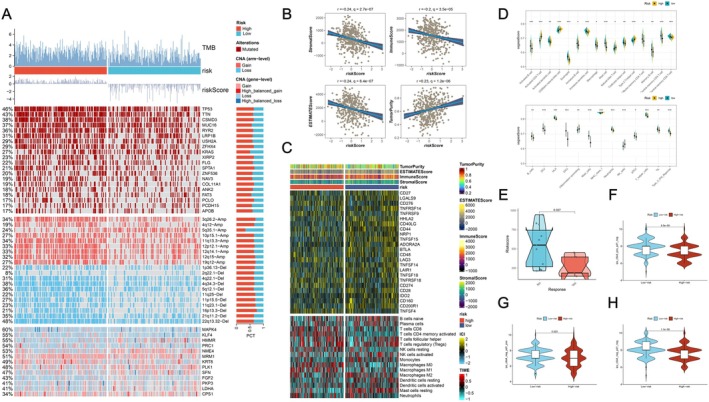
Correlation between genomic variations, tumour immunity and LLPSAS. (A) A comparison of the genomic variation landscape between the high‐ and low‐risk groups is presented in the heatmap. (B) The correlation between the risk score and immune score, stromal score, ESTIMATE score and tumour purity is visualised in the scatter plot. (C) The differences in immune checkpoint expression and immune cell infiltration between the two groups are compared in the heatmap. (D) The TIME is evaluated for differences between the two groups using the ssGSEA algorithm. (E) The association between the LLPSAS risk score and the response to immune therapy is examined in the GSE126044 dataset. (F‐H) Potential benefits of PD‐1 and CTLA‐4 treatments in different risk groups are inferred from the TCIA analysis, which demonstrates differences in IPS scores between the two groups.

Genomic variations and TMB take a pivotal part in tumour bioinformatics analysis, facilitating the establishment of molecular subtyping, prediction of immune therapy response and patient prognosis, as well as identification of tumour driver genes for precision medicine and targeted therapy guidance [35, 36]. Through our analysis, we successfully identified the top 20 genes that harboured the highest mutation frequencies in two risk groups of LLPSAS. Within these groups, TP53, TTN, CSMD3, MUC16, RYR2 and other genes displayed a broader range of mutations, particularly in the high‐risk group. Additionally, enhanced levels of chromosomal instability (CIN) were observed in the high‐risk group (Figure 6A, Figure S8A,B). Among these mutations, missense mutation was the most prevalent type, with a higher prevalence of C>A mutations compared to C>T and C>G conversions (Figure S8C). Subsequently, we calculated TMB scores for the two risk groups, revealing significantly higher scores in the high‐risk group (Figure S8D, *p* < 0.05). For the investigation of the influence of TMB on prognosis, all LUAD patients were grouped into two subsets with high and low TMB respectively. Then, K‐M analysis demonstrated that higher TMB levels positively correlated with improved OS (Figure S8E, *p* < 0.05). Subsequently, for the prognostic prediction of LUAD patients, we combined the TMB score with the risk score to perform additional analyses. According to the median value of the risk score and TMB score, patients were categorised into four groups. The findings of subsequent survival analysis highlighted the most favourable and poorest prognosis in the H‐TMB + low‐risk and L‐TMB + high‐risk groups respectively, thereby emphasising the enhanced prognostic value achieved by combining these two indicators (Figure S8F, *p* < 0.05).

### 3.8 Correlation Between Tumour Immunity and LLPSAS

The tumour immune microenvironment (TIME) is tightly associated with tumour development, where events such as immune surveillance, immune regulation and tumour immune evasion play important roles in early‐stage lung cancer and metastasis [37, 38]. In order to explore the immune status reflected by LLPSAS, an analysis was conducted to investigate the relationship between LLPSAS, immune cells as well as ICs. The immune score, stromal score and ESTIMATE score were calculated for each patient using the ESTIMATE algorithm, enabling a comparison of variations in immune cell infiltration between the two risk groups. The findings revealed the risk score negatively correlated to the immune score, stromal score and ESTIMATE score, while positively correlated to the tumour purity, indicating that patients with low‐risk score might exhibit enhanced levels of immune cell infiltration (Figure 6B,C). Moreover, we found that in the TCGA dataset, ICs including CD27, CD28, CD44, CD48, CD160, CD200R1, CD40LG, TNFSF14, TNFRSF14, TNFSF15, TNFSF18, ADORA2A, BTLA, LGALS9, LAIR1, HHLA2 and IDO2 exhibited enhanced expression levels in patients with low‐risk score (Figure 6C). This implied that patients with low‐risk score might be more likely to benefit from immunotherapy targeting these specific ICs. Subsequently, the assessment of the TIME in the two risk groups was conducted utilising the ssGSEA algorithm. The findings demonstrated that the low‐risk group displayed higher ssGSEA scores in a plethora of immune cells, including B cells, dendritic cells, eosinophils, macrophages, mast cells, T follicular helper cells, and memory T cells (Figure 6D, *p* < 0.05). Additionally, an elevation in scores associated with human leukocyte antigen (HLA), tumour‐infiltrating lymphocytes (TILs) and type II interferon response was observed in the low‐risk group (Figure 6D, *p* < 0.05). Recently, the comprehensive treatment of advanced‐stage tumours has increasingly relied on ICB agents targeting PD‐1/PD‐L1, CTLA‐4 [39]. To further explore the potential role of LLPSAS in immunotherapies, we utilised the GSE126044 dataset, which comprised transcriptomic data from 16 NSCLC patients treated with anti‐PD‐1 agents and including responders and non‐responders, to calculate the LLPSAS risk score. Surprisingly, we found that patients with low‐risk scores exhibited a more favourable response to immunotherapy (Figure 6E, *p* < 0.05). Additionally, the IPS of LUAD patients was acquired from the TCIA database to estimate their immunogenicity and predict their potential response to ICB agents. We found an elevated IPS score in the low‐risk group, indicating that this group may demonstrate a more favourable response to ICB (Figure 6F–H). In summary, the low‐risk group demonstrated a more robust immune response and enhanced anti‐tumour immune capabilities, making them potentially benefit from immunotherapy, which may lead to more favourable prognostic outcomes.

### 3.9 Prediction of Potential Effective Small‐Molecule Drugs and Drug Sensitivity

**FIGURE 7:**
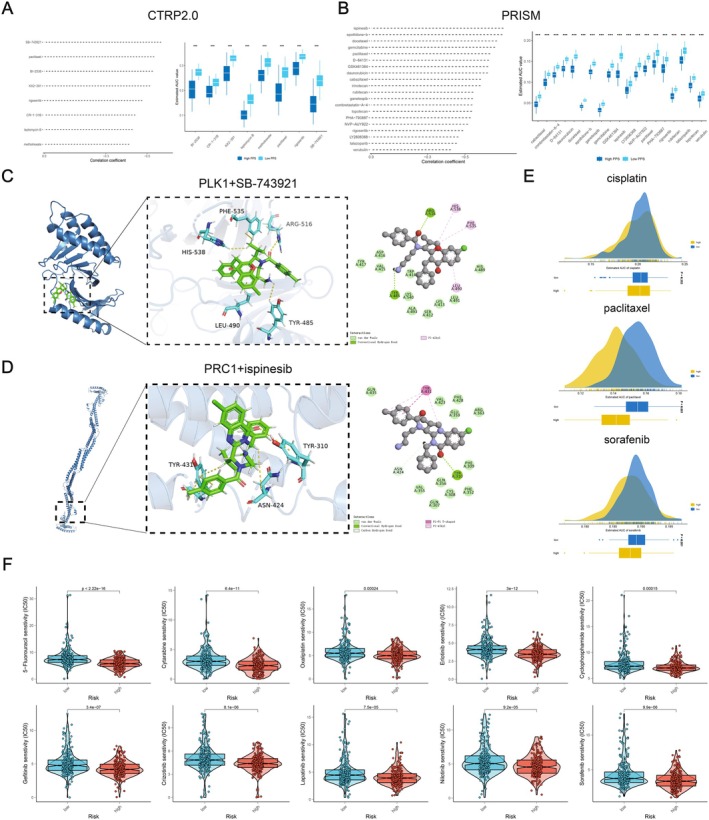
Prediction of potential effective small‐molecule drugs and drug sensitivity. (A, B) A total of 8 CTRP‐derived and 20 PRISM‐derived compounds were obtained from drug resistance prediction models based on the CTRP2.0 (A) and PRISM (B) datasets. (C) The molecular docking model illustrates the interaction between the PLK1 protein and the small molecule drug SB‐743921. (D) The molecular docking model demonstrates the interaction between the PRC1 protein and the small molecule drug ispinesib. (E) The drug sensitivity of three frequently utilised clinical drugs, namely cisplatin, paclitaxel and sorafenib, was assessed. (F) The IC50 values for commonly used drugs of chemotherapy and targeted therapy were compared between the two risk groups.

Considering that chemotherapy combined with targeted therapy remains the standard treatment for advanced‐stage NSCLC [39], we used the CTRP2.0 and PRISM databases to predict potentially effective small molecule drugs, aiming to improve treatment response and prognosis in the high‐risk group. Based on the Spearman correlation between risk scores and AUC scores, we identified 8 CTRP2.0 derivatives (including SB‐743921, paclitaxel, B‐2536, KX2‐391, rigosertib, CR‐1‐31B, leptomycin B, methotrexate) and 20 PRISM derivatives (including ispinesib etc.) with Spearman's *r* < −0.35 and *p* < 0.05. We found that in the high‐risk group, all 28 derivatives exhibited lower AUC values (Figure 7A,B). Subsequently, we selected SB‐743921 and ispinesib as two drugs and performed molecular docking using software such as MOE 2019 on the proteins encoded by PLK1, HMMR and PRC1 (the three modelling genes) (Figure 7C,D, Figure S9). The docking scores between PLK1, HMMR, PRC1 proteins and SB‐743921, ispinesib were −6.2504 kcal/mol, −6.8400 kcal/mol (PLK1), −5.2226 kcal/mol, −5.2136 kcal/mol (HMMR), −5.3946 kcal/mol, −5.6303 kcal/mol (PRC1), respectively. Lower binding energies in molecular docking indicate stronger binding affinity between the compound and the protein target. The docking scores between these two drugs and the three modelling genes were all less than −5 kcal/mol, suggesting a perfect interaction between the drugs and the modelling genes. The binding between these small molecule compounds and the target proteins is mainly mediated by hydrogen bond interactions and hydrophobic interactions. For example, Tyr485 and Arg516 residues in the PLK1 protein form hydrogen bond interactions with SB‐743921, while His538, Phe535 and Leu490 residues form hydrophobic interactions with SB‐743921 (Figure 7C). Cisplatin, paclitaxel and sorafenib, three commonly used clinical drugs, have been shown to be more effective in high‐risk patients (Figure 7E). Cisplatin is a platinum‐based compound that primarily interferes with DNA replication and cell division of cancer cells by binding to DNA [40]. Consistency with the results of drug sensitivity prediction can be observed in the positive correlation between the LLPSAS risk score and oncogenic pathways associated with DNA replication and cell cycle regulation, as mentioned in the previous enrichment analysis. To validate these findings, we proceeded to calculate the IC50 of kinds of common anti‐tumour drugs by the use of the GDSC database. The results revealed that the high‐risk group exhibited significantly elevated IC50 values for chemotherapy drugs (such as 5‐fluorouracil, oxaliplatin, cyclophosphamide) and targeted therapy drugs (such as sorafenib, gefitinib, erlotinib). This finding suggested that patients with a high‐risk score might more favourably response to these drugs (Figure 7F), which could potentially compensate for the observed poor response to immune therapy in this group.

### 3.10 Experimental Validation of the Key Genes in the LLPSAS

The results of our bioinformatics analyses showed the outstanding predictive performance of LLPSAS in LUAD patients' outcomes and treatment response. We evaluated the correlation between 13 key genes and the LLPSAS using ROC curves. We identified the top three genes most closely associated with the LLPSAS, namely PLK1 (AUC = 0.957), HMMR (AUC = 0.942) and PRC1 (AUC = 0.869) (Figure S10A). Utilising transcriptomic data from the TCGA and GTEx databases, we conducted an assessment of the differential expression of these 13 modelling genes in LUAD as compared to normal lung tissues. The findings revealed that PLK1, HMMR and PRC1 were relatively highly expressed in LUAD (Figure S10B). These findings were further validated using immunohistochemistry (IHC) images obtained from the Human Protein Atlas (HPA) database (Figure S11).

**FIGURE 8:**
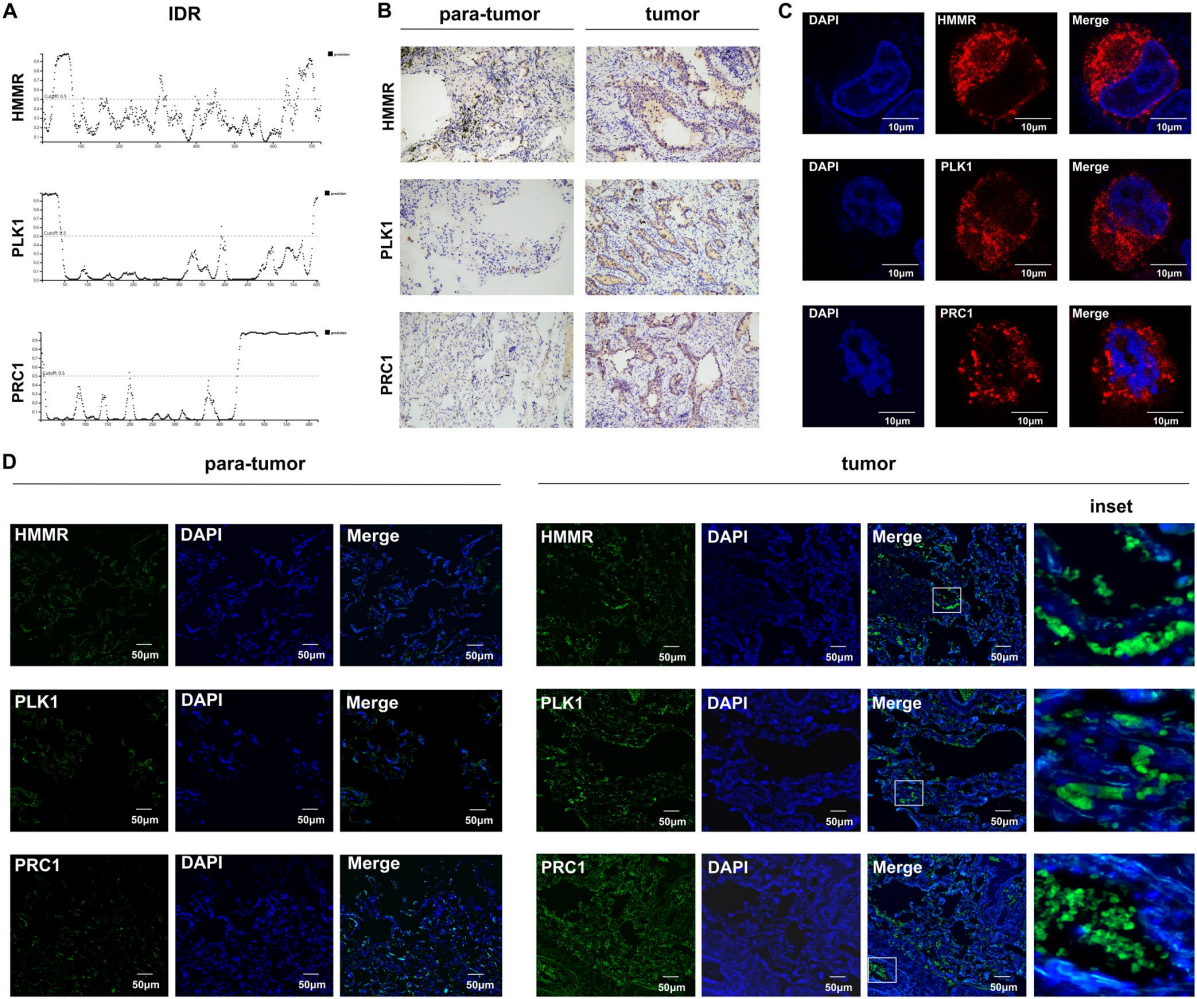
Experimental validation of the key genes in the LLPSAS. (A) The intrinsic disorder regions (IDRs) of the proteins HMMR, PLK1 and PRC1 were visualised using the PSIPRED website. (B) IHC staining of HMMR, PLK1 and PRC1 in tumour sections and para‐tumour sections; images were captured at 20×. (C) Immunofluorescence staining of HMMR, PLK1 and PRC1 was performed in SPC‐A‐1 cells. (D) Immunofluorescence staining of HMMR, PLK1 and PRC1 in tumour sections and para‐tumour sections; representative images of the existence of fluorescent foci on these genes were showed in inset, respectively.

The multivalent interactions between intrinsically disordered regions (IDRs) of protein or RNA molecules are fundamental prerequisites for the occurrence of LLPS within cells, where these interactions drive their aggregation and formation of condensates. To further explore the ability of these three key genes (PLK1, HMMR and PRC1) to undergo LLPS in LUAD cells, we visualised the IDRs of the protein translated from these three genes using the PSIPRED website (http://bioinf.cs.ucl.ac.uk/psipred/?disopred=1). The results demonstrated that all three target protein sequences contained IDR regions (Figure 8A, Figure S12). The expression of these three key genes was determined using an IHC experiment, and it was showed that these genes exhibited significantly higher expression in the tumour tissues compared to the para‐tumour tissues (Figure 8B). Then, we further validated the presence of fluorescent foci in the target proteins using tissue and cellular immunofluorescent staining. The results showed that fluorescent foci were observed in both tumour sections and LUAD cells upon staining of the target proteins, while they were rarely seen in the para‐tumour tissues (Figure 8C,D). Collectively, these findings showed the overexpression and LLPS association of key genes engaged in our LLPSAS model, which were consistent with our previous bioinformatic results, providing reliable support for fundamental construction of our LLPSAS model.

## 4 Discussion

Human health is greatly jeopardised by lung cancer, which ranks among the most prevalent malignant tumours globally [1]. In addition to traditional surgical treatment and chemotherapy, targeted therapies represented by EGFR inhibitors, ALK inhibitors, ROS1 inhibitors and immunotherapies represented by PD‐1/PD‐L1 inhibitors and CTLA‐4 inhibitors are playing an increasingly critical role in the treatment of lung cancer [3, 4, 5]. Nevertheless, despite notable advancements in the realm of lung cancer treatment, the overall therapeutic outcomes and prognosis for most advanced LUAD patients continue to be unsatisfactory. In recent years, the rapid progress of bulk RNA‐seq and scRNA‐seq technologies has facilitated the identification of numerous tumour markers and therapeutic targets in lung cancer. As a result, an expanding repertoire of diagnostic and prognostic signatures has been constructed with the aim of enhancing the prognosis of individuals afflicted with this disease.

In recent years, as our understanding of the mechanisms underlying lung cancer has deepened, researchers have found that LLPS takes a crucial part in lung cancer, participating in a series of tumour biological processes including gene expression regulation, DNA damage and repair, metabolic regulation and intracellular signalling [23], and is closely related to targeted therapies in lung cancer [41], which may ultimately impact the prognosis of LUAD patients. However, surprisingly, we have found that LLPS‐related signatures are remarkably limited in the existing prognostic signatures for LUAD. Consequently, the objective of this investigation involves the development of an LLPS‐associated signature (LLPSAS) in LUAD through the combination of scRNA‐seq and bulk RNA‐seq technologies. This comprehensive approach enables the utilisation of the LLPSAS for predicting the prognosis and treatment response of LUAD patients. Moreover, machine learning algorithms, based on the foundations of statistics and computer science in the field of artificial intelligence, extract patterns, trends and correlations from large datasets through training and learning processes, and can be applied to tasks such as prediction, classification and clustering [42]. Consequently, the initial step of this investigation involved the analysis of scRNA‐seq data from lung adenocarcinoma to identify LLPS‐related genes within the single‐cell dataset. Furthermore, a comparison was made to ascertain the dissimilarities in cell communication between the high and low LLPS‐AUC groups. Following this, a robust prognostic signature, LLPSAS, was developed for LUAD using bulk RNA‐seq data and a combination of 101 machine learning algorithms, leveraging the LLPS‐related genes.

LLPSAS consists of 13 LLPS‐related genes, including CPS1, FGF2, HMMR, KLF4, KRT8, LDHA, MAPK4, MRM1, NME4, PKP3, PLK1, PRC1 and SFN, with most of these genes playing a pro‐oncogenic role. An example of this is the regulation of Carbamoyl phosphate synthetase 1 (CPS1) in the cell urea cycle as the rate‐limiting enzyme, and the high expression of CPS1 is tightly associated with kinds of tumours including LUAD, hepatocellular carcinoma (HCC), gastric cancer (GC) and small intestinal adenocarcinoma [43, 44, 45]. Fibroblast growth factor 2 (FGF2), a growth factor produced by carcinoma‐associated fibroblasts, is reported to be involved in the proliferation, migration and chemotherapy resistance of tumour cells [46, 47]. In lung cancer cells, it has been demonstrated that targeting FGF2 could inhibit the cell growth mediated by the FGF2‐FEFR pathway [48]. Hyaluronan mediated motility receptor (HMMR) also plays a multiple role in cell proliferation, migration and differentiation [49]. In HCC, HMMR was reported to might be a potential target owe to its regulation of autophagy in tumour progression [50]. In LUAD, HMMR was significantly expressed in tumour tissues and closely associated with patient survival and TME. Krüppel‐like factor 4 (KLF4) is one of the Kruppel‐like factors that could serve as a multifunctional player participating in the development and progression of multiple cancers [51]. Despite the ambivalent nature of KLF4 in tumorigenesis, it was conveyed that KLF4 could suppress LUAD development [52, 53]. The glycolytic enzyme lactate dehydrogenase A (LDHA) is also closely associated with numerous tumours including pancreatic cancer, GC, HCC, breast cancer and lung cancer [54]. Inhibition of LDHA expressed significance in suppression of tumour progression and improvement of sensitivity for chemotherapy as well as radiotherapy [54]. Mitogen‐Activated Protein Kinases 4 (MAPK4), a non‐classic MAPK, is highly expressed in kinds of malignancies, such as prostate cancer, breast cancer and cervical cancer [55]. Interestingly, MAPK4 was demonstrated to promote tumours through direct and specific activation of AKT/mTOR via an alternative pathway without dependence on PI3K/PDK1 [56]. In addition, stratifin (SFN), a cell cycle checkpoint protein and a regulator of mitotic translation that is closely related to DNA damage, is reported to facilitate lung cancer development and progression [57, 58]. Polo‐like kinase 1 (PLK1) also participates in the regulation of the cell cycle, where it assumes a pivotal role in the initiation, sustenance and culmination of mitosis [59]. Notably, PLK1 exhibited overexpression in diverse cancer types and is related to the tumorigenesis and progression in multiple cancers. PLK1 inhibitors are considered promising potential targeted anticancer drugs, and some PLK1 inhibitors have entered clinical trials [60]. Cell movement and accurate cell division rely significantly on the protein regulator of cytokinesis (PRC1). The disruption of PRC1 function results in impairments in cell movement, which in turn facilitates CIN, ultimately contributing to tumour heterogeneity and the progression of cancer [61].

Utilising these 13 genes, the risk score was computed for each sample, and subsequently, all patients were categorised into two groups with different risk‐score levels. Subsequent survival analysis unveiled a poorer OS in the high‐risk group, potentially attributable to the heightened activity of pro‐oncogenic pathways observed within this group. These pathways encompassed DNA replication, cell cycle regulation, EMT, glycolysis, oxidative phosphorylation and the PI3K‐AKT–mTOR signalling pathway. Comparative analysis of AUC and C‐index demonstrated that LLPSAS exhibited superior prognostic value in comparison with clinical features such as age, gender and stage. To further ascertain the predictive efficacy of LLPSAS, a comparison was conducted against 140 published signatures from the TCGA, GSE30219, GSE31210, GSE42127 and GSE68465 datasets. Residual heterogeneity was observed across multiple datasets even after batch effect correction. To enhance reliability and demonstrate model generalisability, validation was performed using independent datasets. Consequently, the TCGA‐LUAD dataset served as the training set, while the GSE30219, GSE31210, GSE42127 and GSE68465 datasets constituted the validation sets. Encouragingly, the results indicated that LLPSAS outperformed all other 140 previously published signatures in each dataset. Considering the limitations of purely bioinformatics analysis, we performed experimental validation on three genes, HMMR, PLK1 and PRC1, which were most closely associated with the model. The results of IHC confirmed significant upregulation of protein expression for the above three genes in LUAD tissues, consistent with the analysis results from the database. Additionally, we performed immunofluorescence staining to validate the formation of fluorescent foci involving these three proteins in both LUAD tumour sections and cells.

The LLPSAS signature developed in this study demonstrated significant association with LUAD prognosis, exhibiting superior predictive performance and notable immune relevance. These characteristics highlight its translational potential as a clinical biomarker. Integration of this signature into clinical practice may support personalised risk stratification, ultimately improving patient outcomes. However, there are still many questions to be addressed. Firstly, the reliance on public datasets introduces potential selection biases, which may impact gene expression patterns and limit the comprehensiveness of our findings. Secondly, the generalisability of our LLPSAS across diverse populations and clinical settings requires further validation, as cohort heterogeneity remains a challenge. To address these, on the one hand, multi‐centre prospective studies need to be conducted to robustly validate the prognostic reliability of LLPSAS. On the other hand, functional screens (e.g., in vitro and in vivo experiments) could be performed to elucidate the precise biological roles of the identified 13 LLPS‐related genes in LUAD.

In summary, by integrating scRNA‐seq and bulk RNA‐seq data, coupled with the utilisation of a fusion of 101 machine learning algorithms, we successfully developed a highly promising LLPS‐associated signature (LLPSAS) in LUAD. This signature exhibited the ability for predicting prognosis and treatment response in patients. The emergence of LLPSAS provides further insights into the involvement of LLPS in LUAD and offers the potential to improve the outcomes of LUAD patients.

## Author Contributions

**Zipei Song:** conceptualization (lead), data curation (lead), formal analysis (lead), project administration (lead), validation (lead), visualization (lead), writing – original draft (lead), writing – review and editing (lead). **Yuting Li:** conceptualization (equal), validation (equal), visualization (equal), writing – original draft (equal), writing – review and editing (equal). **Pengpeng Zhang:** conceptualization (equal), project administration (equal), writing – original draft (equal), writing – review and editing (equal). **Ke Wei:** conceptualization (equal), writing – original draft (equal), writing – review and editing (equal). **Miaolin Zhu:** validation (lead), visualization (lead), writing – original draft (equal), writing – review and editing (equal). **Yuheng Wang:** writing – original draft (equal), writing – review and editing (equal). **Zhihua Li:** conceptualization (equal). **Liang Chen:** conceptualization (lead), funding acquisition (lead), project administration (lead), resources (lead), writing – review and editing (lead). **Jianan Zheng:** writing – review and editing (equal).

## Conflicts of Interest

The authors declare no conflicts of interest.

## Supporting information


**Figure S1:** jcmm70807‐sup‐0001‐FigureS1.pdf.

**Figure S2:** jcmm70807‐sup‐0002‐FigureS2.pdf.

**Figure S3:** jcmm70807‐sup‐0003‐FigureS3.pdf.

**Figure S4:** jcmm70807‐sup‐0004‐FigureS4.pdf.

**Figure S5:** jcmm70807‐sup‐0005‐FigureS5.pdf.

**Figure S6:** jcmm70807‐sup‐0006‐FigureS6.pdf.

**Figure S7:** jcmm70807‐sup‐0007‐FigureS7.pdf.

**Figure S8:** jcmm70807‐sup‐0008‐FigureS8.pdf.

**Figure S9:** jcmm70807‐sup‐0009‐FigureS9.pdf.

**Figure S10:** jcmm70807‐sup‐0010‐FigureS10.pdf.

**Figure S11:** jcmm70807‐sup‐0011‐FigureS11.jpg.

**Figure S12:** jcmm70807‐sup‐0012‐FigureS12.pdf.

**Table S1:** jcmm70807‐sup‐0013‐TableS1.xlsx.

**Table S2:** jcmm70807‐sup‐0014‐TableS2.xlsx.

## Data Availability

The datasets analysed in the current study are available in the TCGA repository (https://portal.gdc.cancer.gov/) and GEO (https://www.ncbi.nlm.nih.gov/geo/). Further inquiries can be directed to the corresponding authors.
