# Supplementary figures and images for: Investigating Liquid–Liquid Phase Separation in Lung Adenocarcinoma to Improve Prognostic Accuracy and Treatment Efficacy
Source: J Cell Mol Med. 2025 Aug 22;29(16):e70807. doi: 10.1111/jcmm.70807 (PMC12372984; doi:10.1111/jcmm.70807)

A

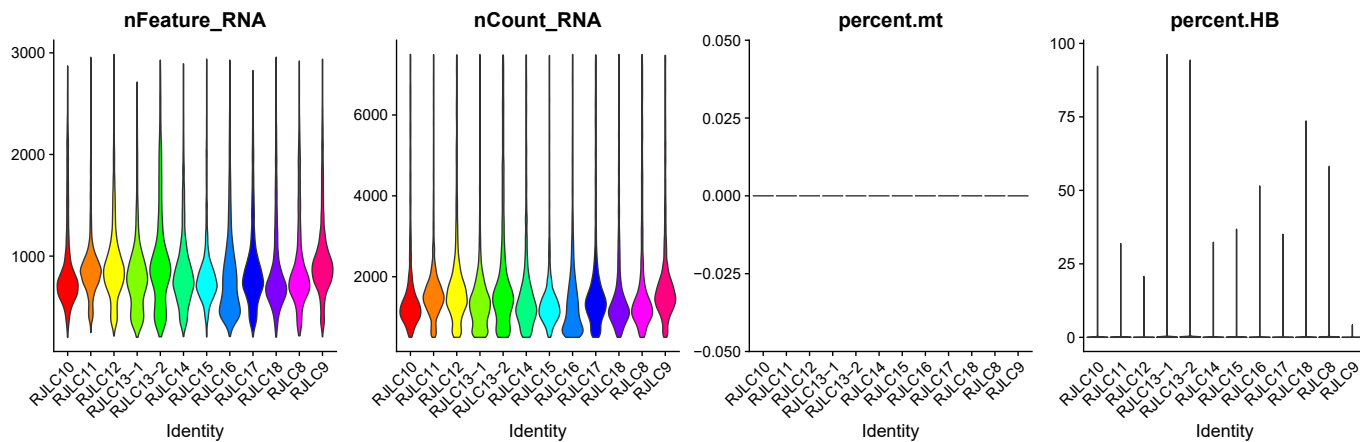

**B**

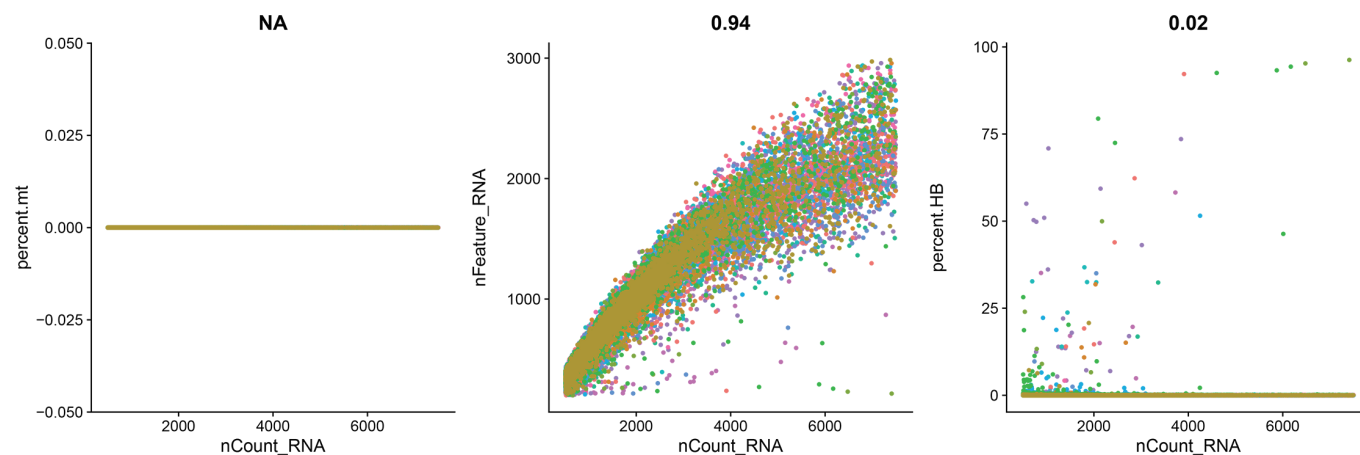

C

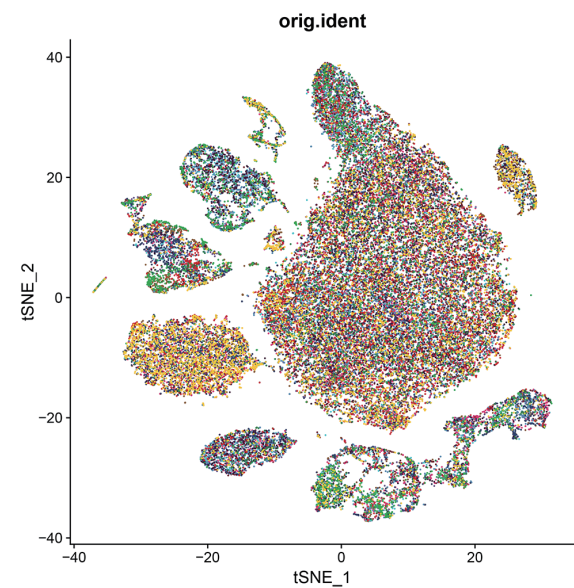

D

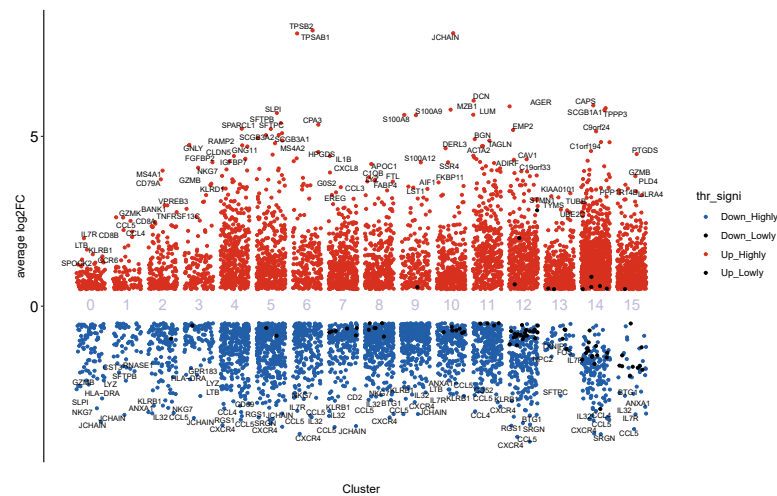

Supplement: Supplementary file 1 — Figure S1: jcmm70807‐sup‐0001‐FigureS1.pdf. [file JCMM-29-e70807-s014.pdf]

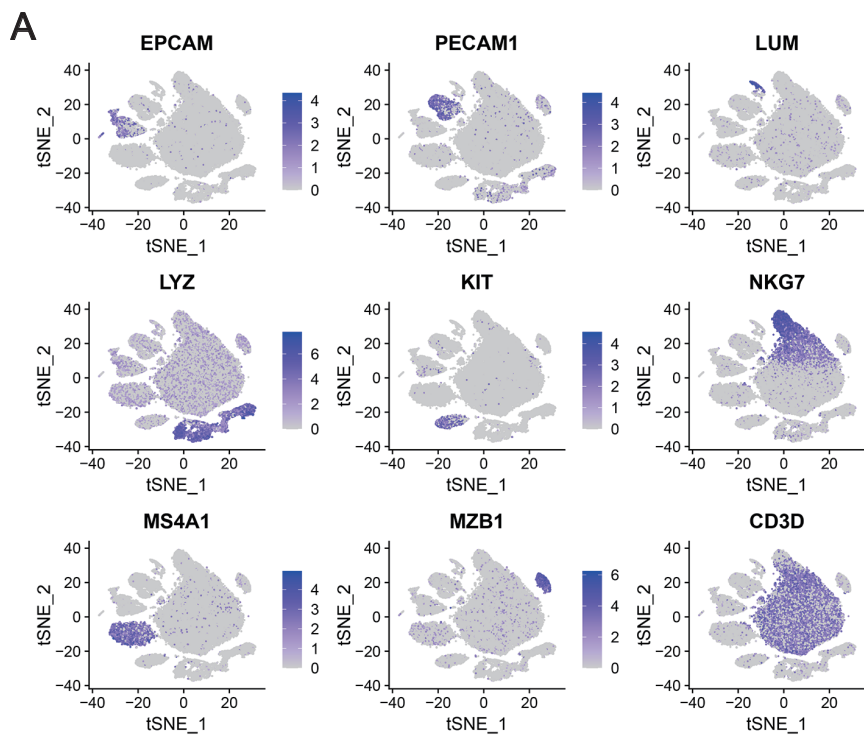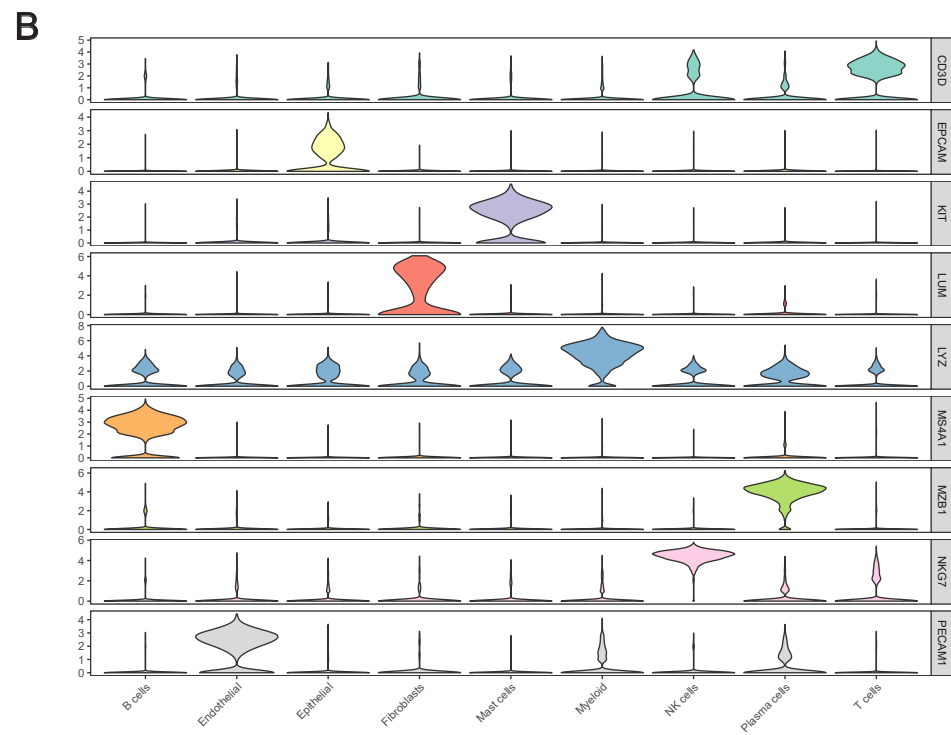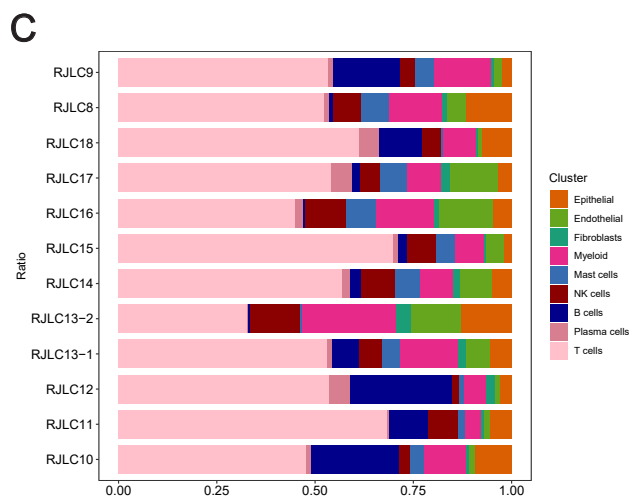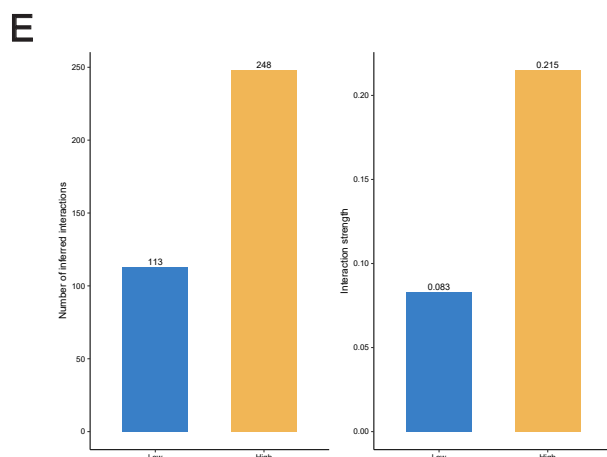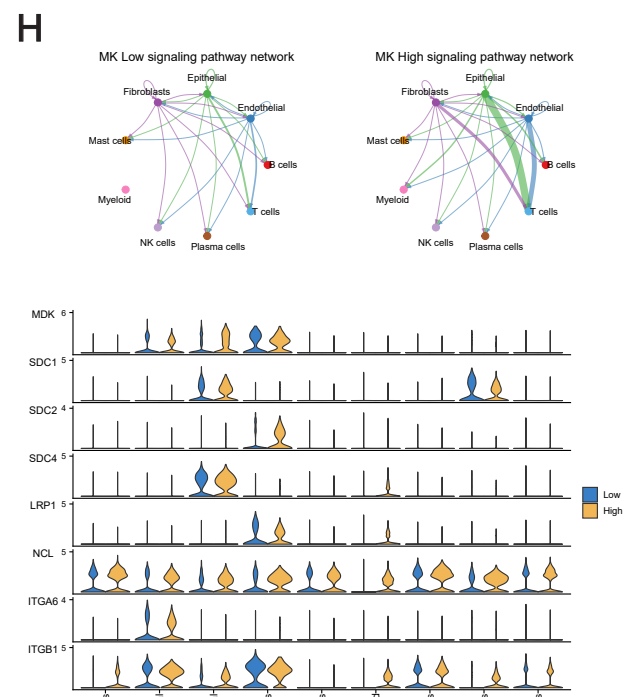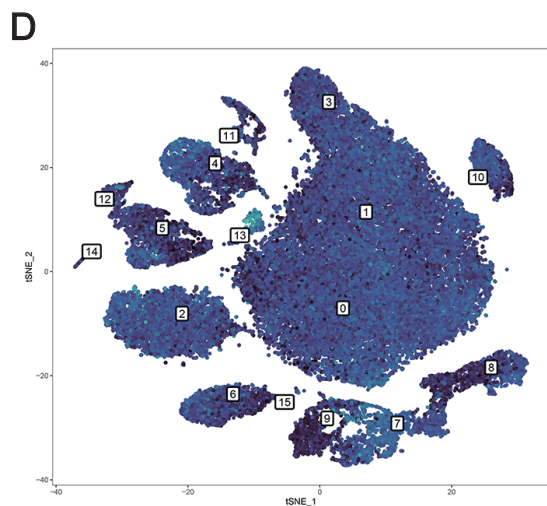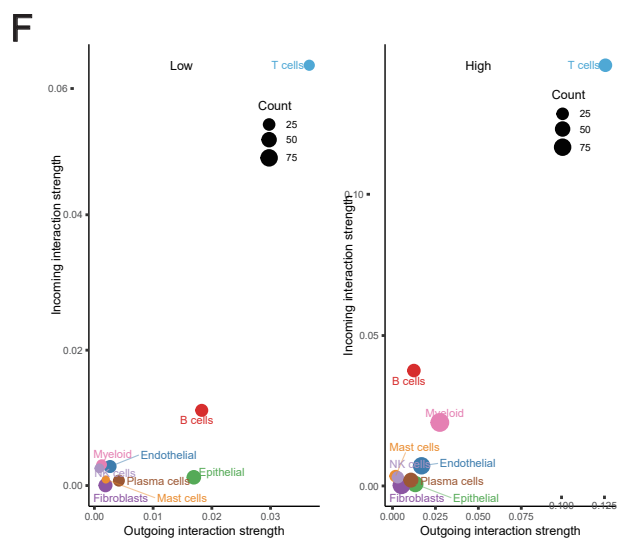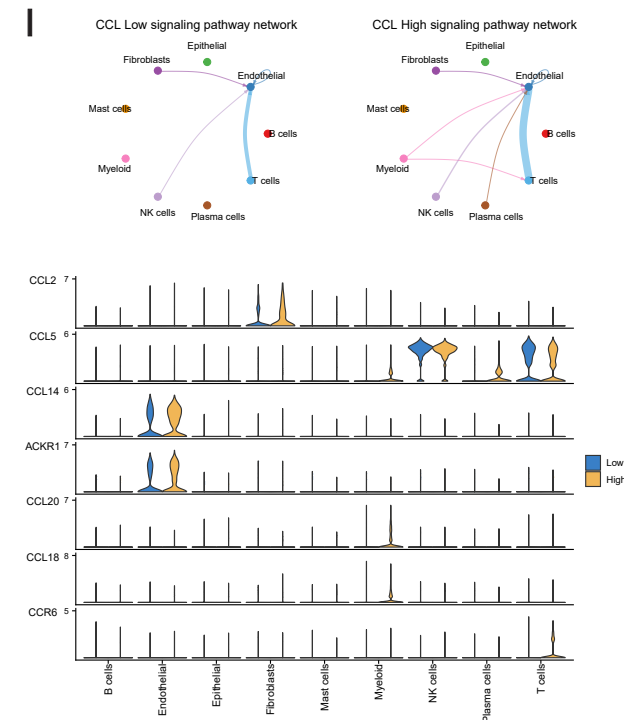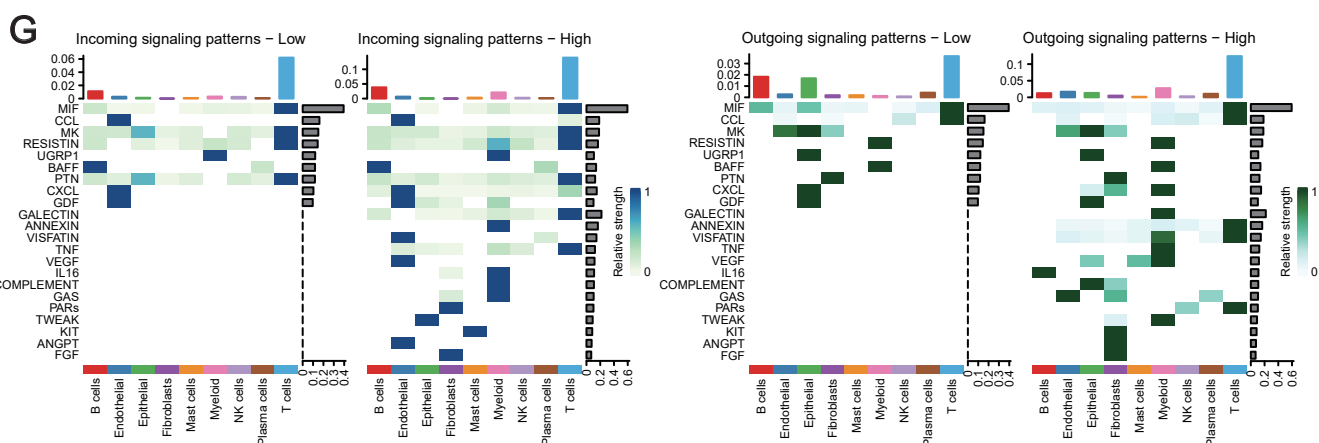

Supplement: Supplementary file 2 — Figure S2: jcmm70807‐sup‐0002‐FigureS2.pdf. [file JCMM-29-e70807-s007.pdf]

A

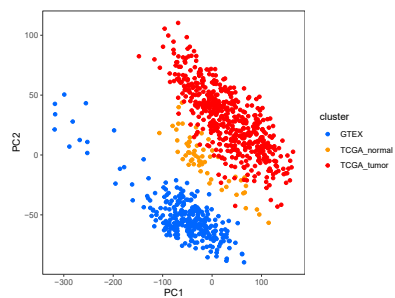

B

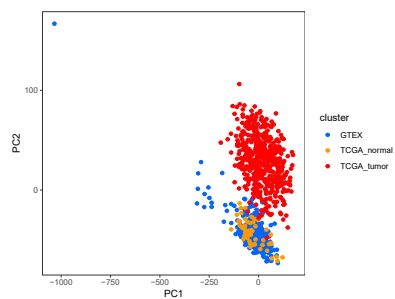

C

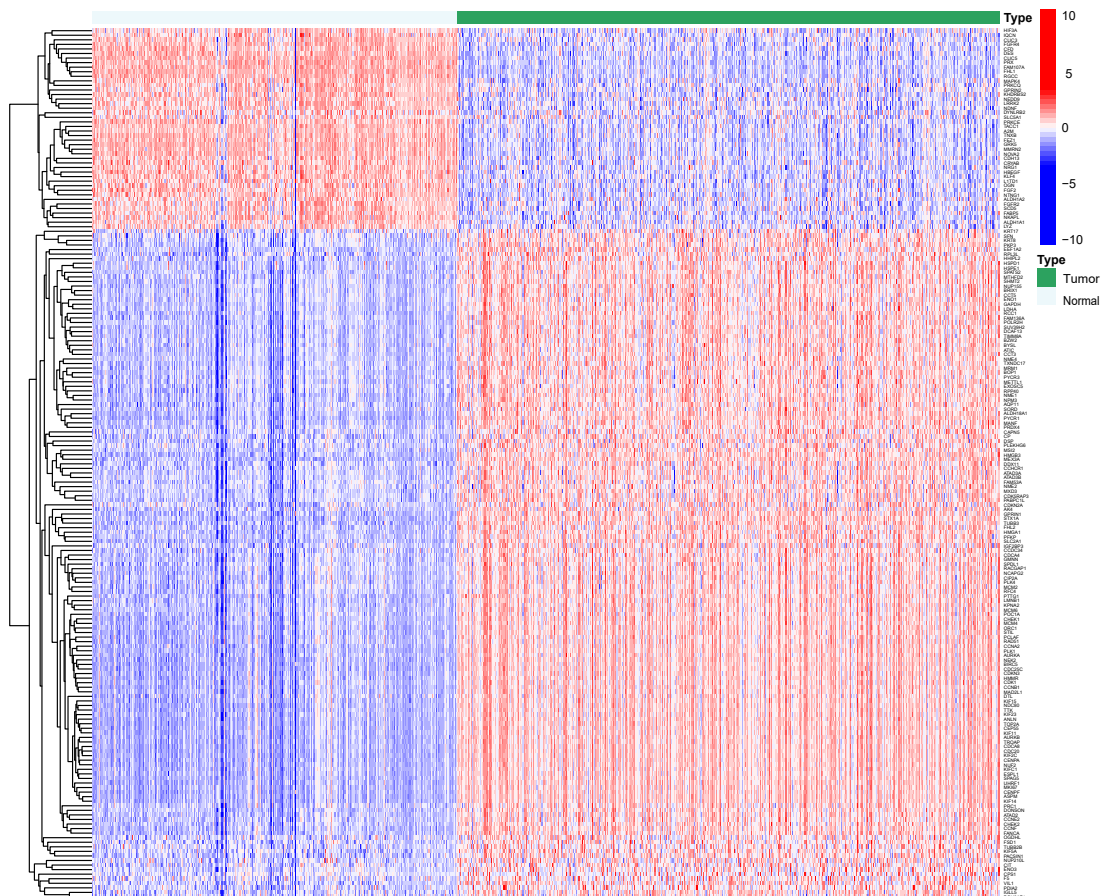

D

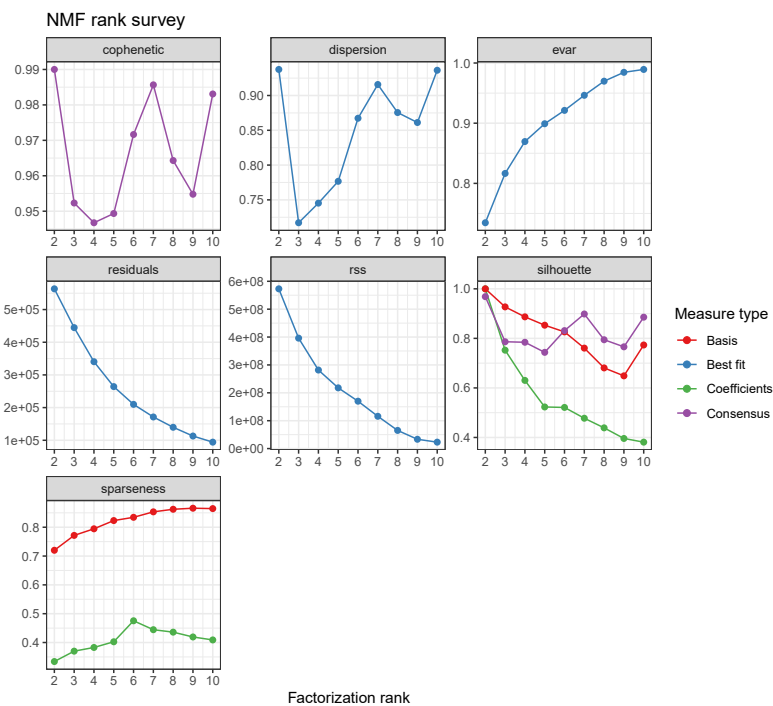

E

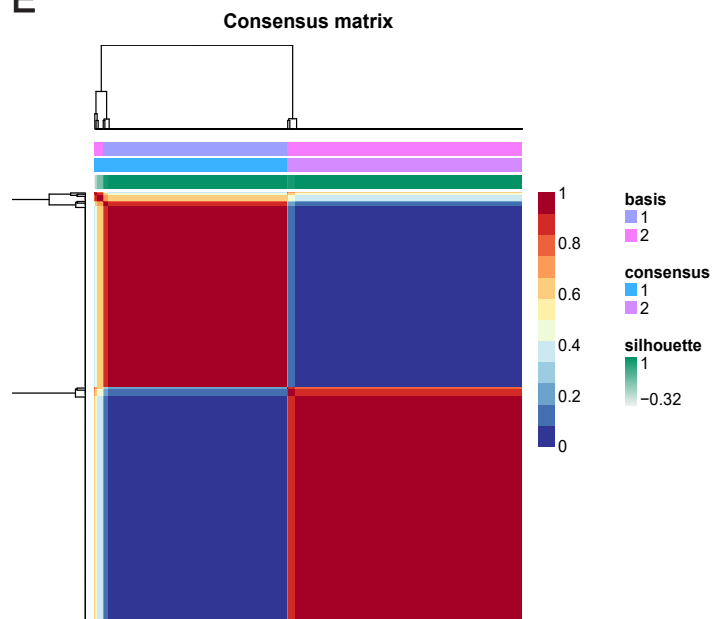

Supplement: Supplementary file 3 — Figure S3: jcmm70807‐sup‐0003‐FigureS3.pdf. [file JCMM-29-e70807-s012.pdf]

A

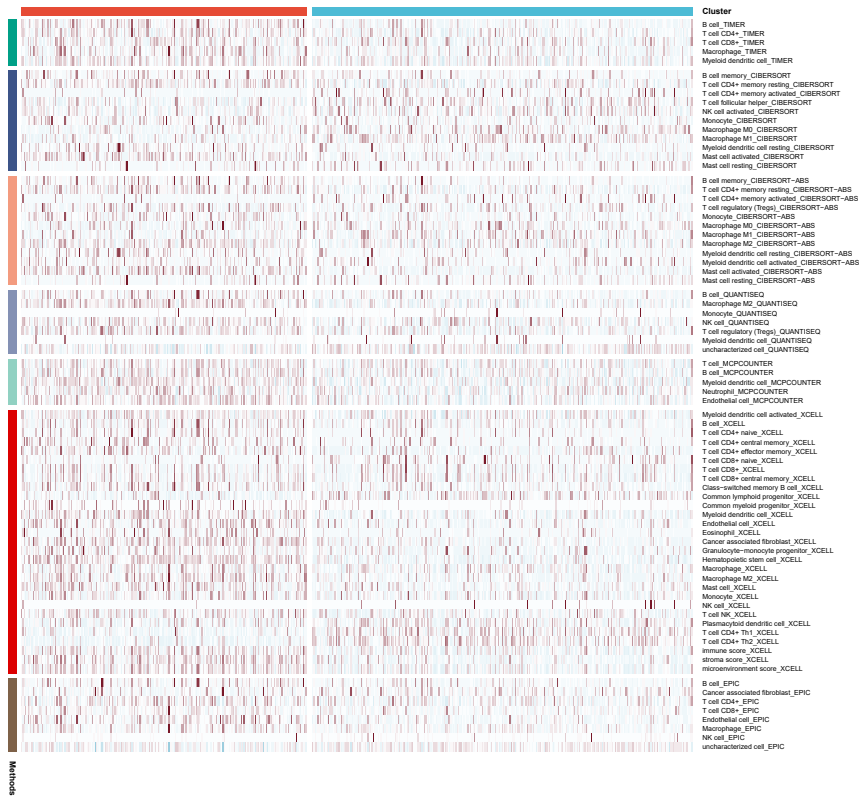

B

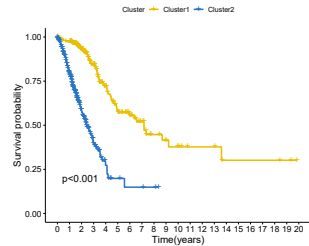

C

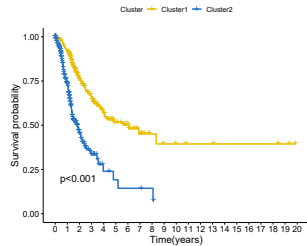

D

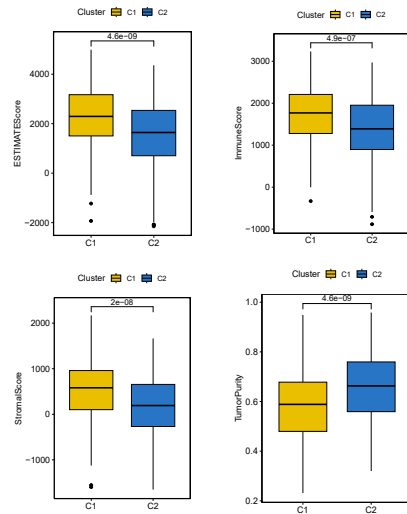

Supplement: Supplementary file 4 — Figure S4: jcmm70807‐sup‐0004‐FigureS4.pdf. [file JCMM-29-e70807-s003.pdf]

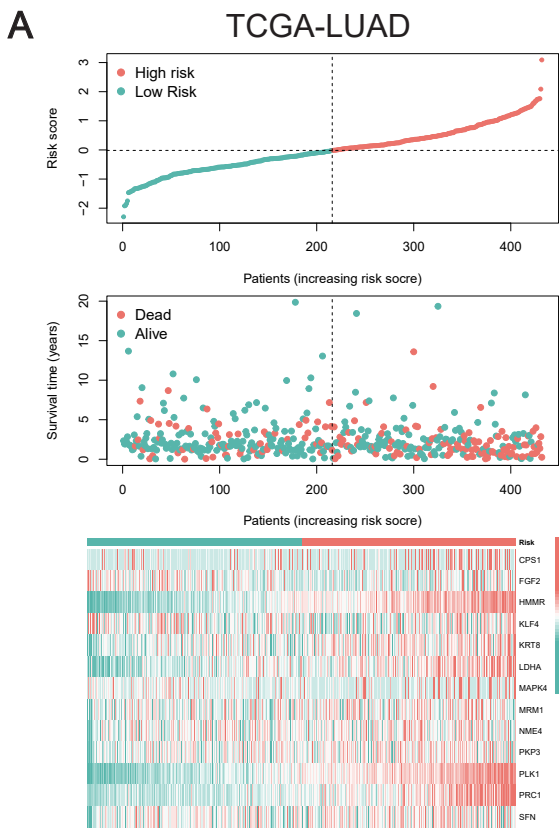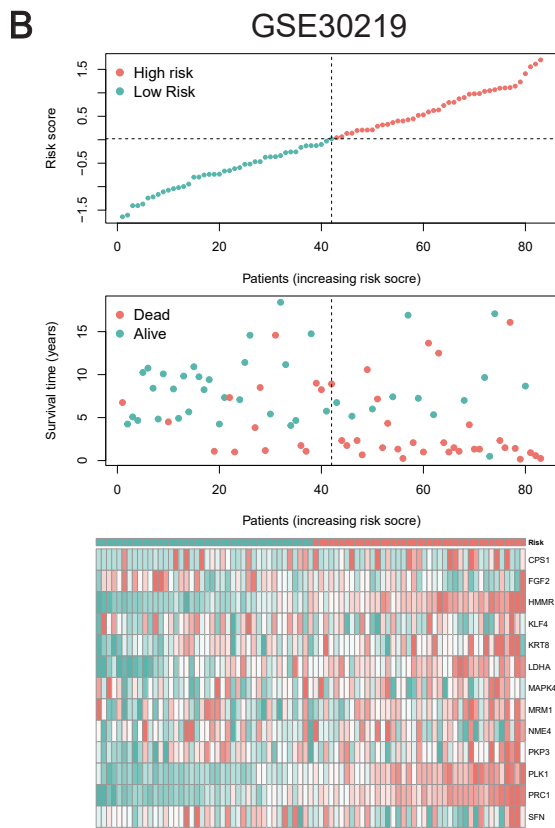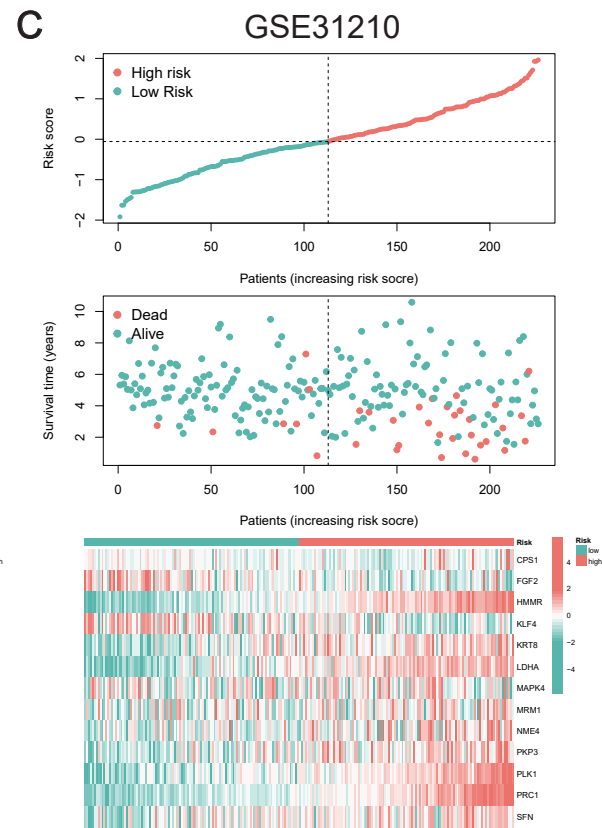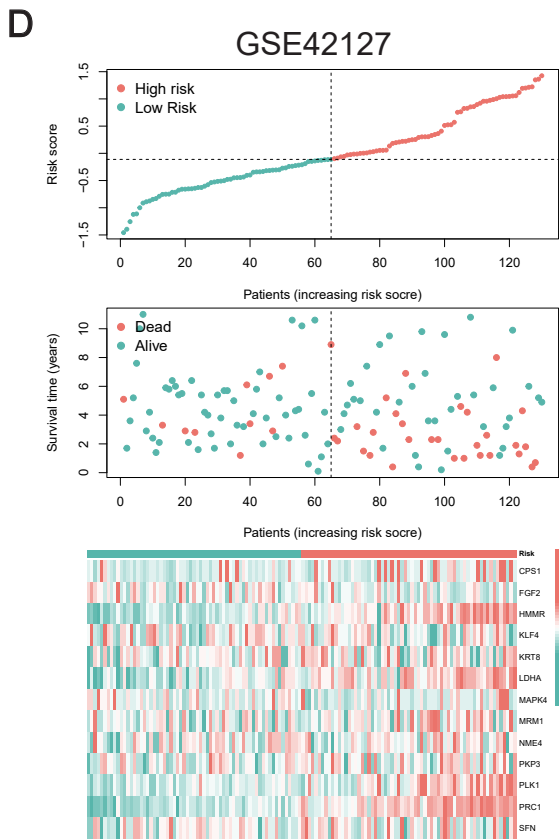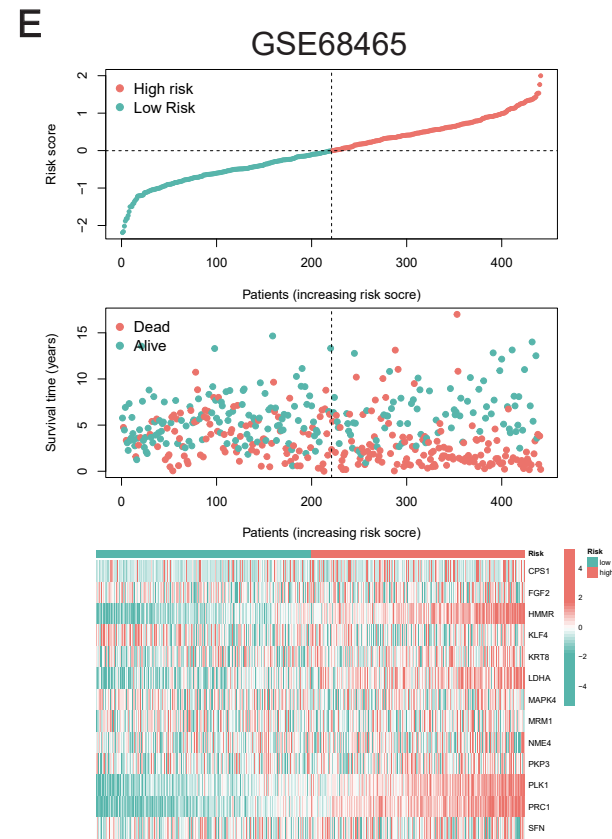

Supplement: Supplementary file 5 — Figure S5: jcmm70807‐sup‐0005‐FigureS5.pdf. [file JCMM-29-e70807-s008.pdf]

A

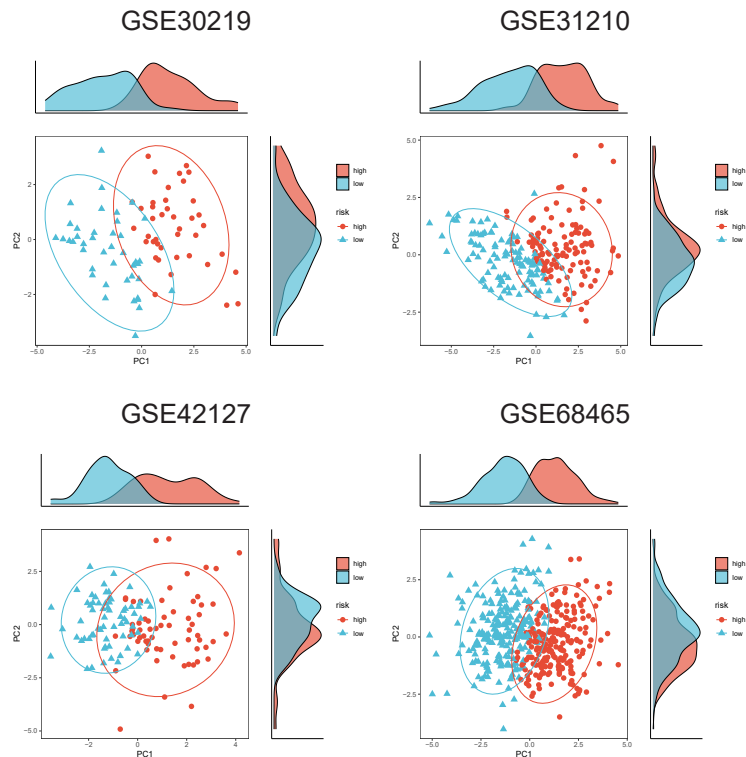

B

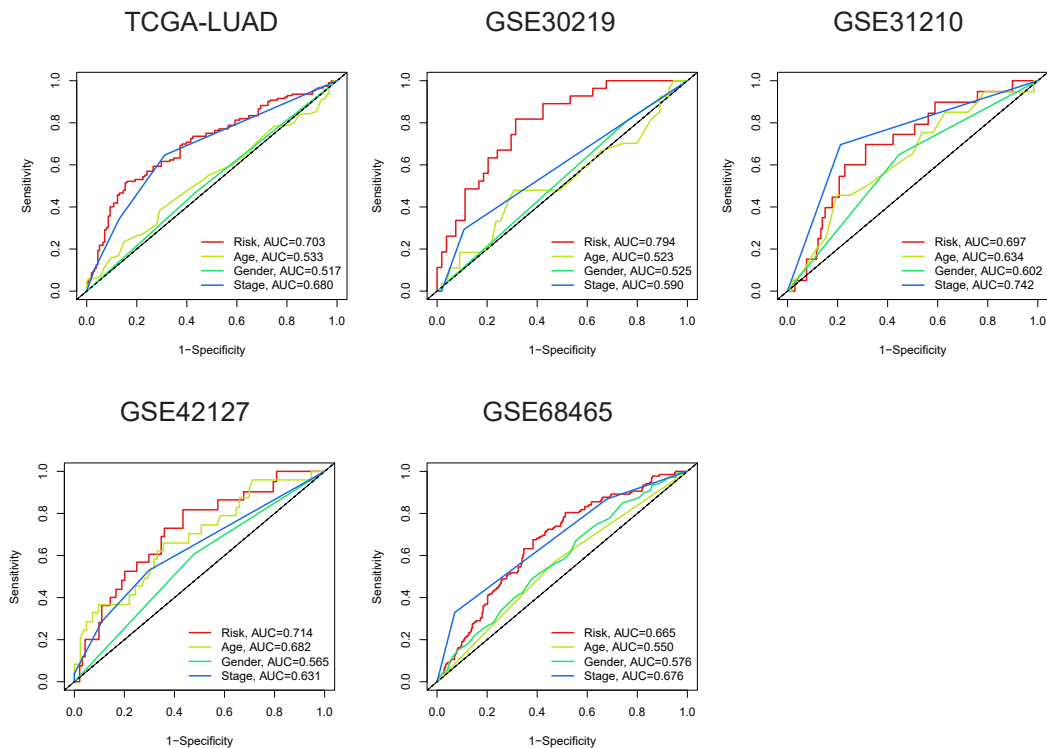

Supplement: Supplementary file 6 — Figure S6: jcmm70807‐sup‐0006‐FigureS6.pdf. [file JCMM-29-e70807-s009.pdf]

A

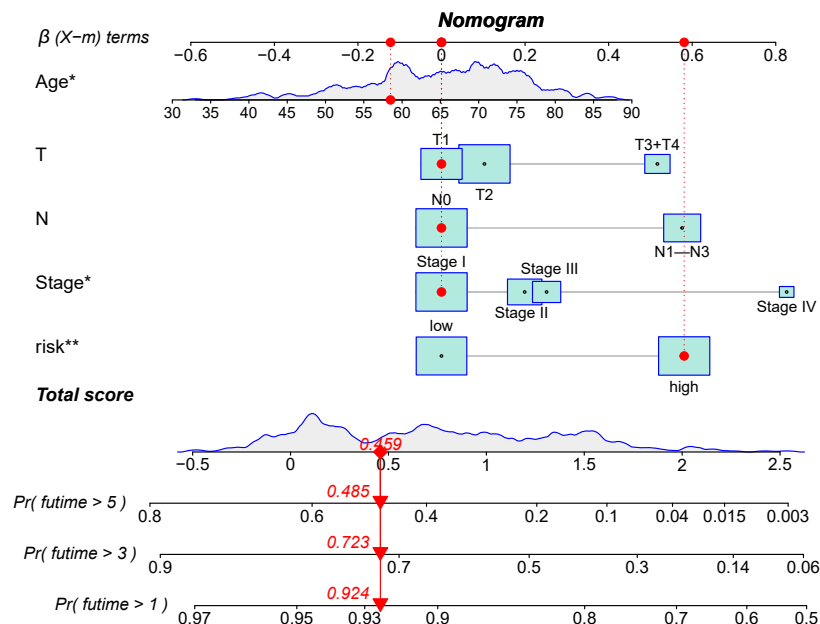

C

TCGA-LUAD

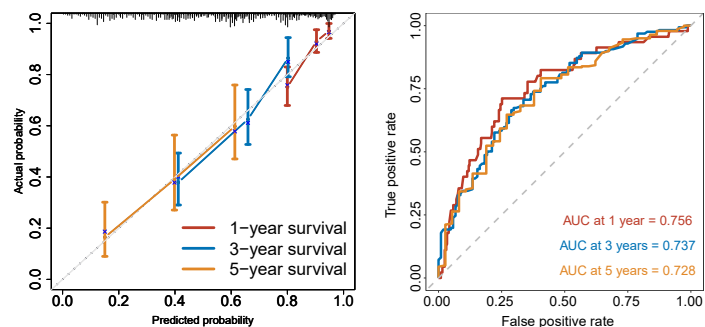

B

forest map

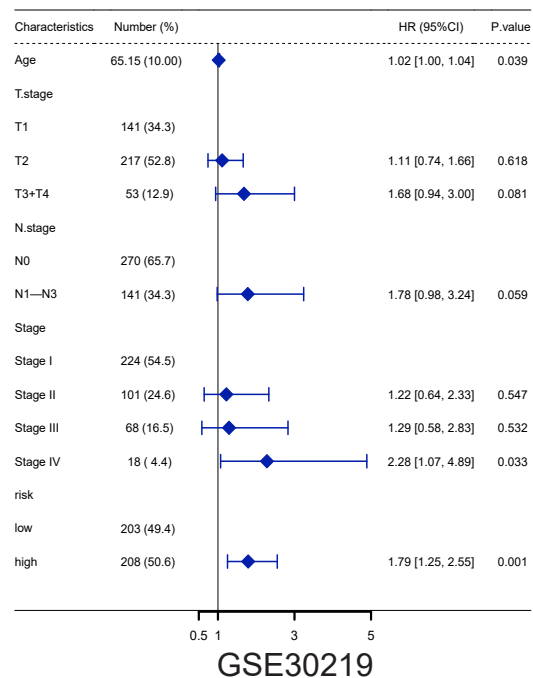

D

GSE30219

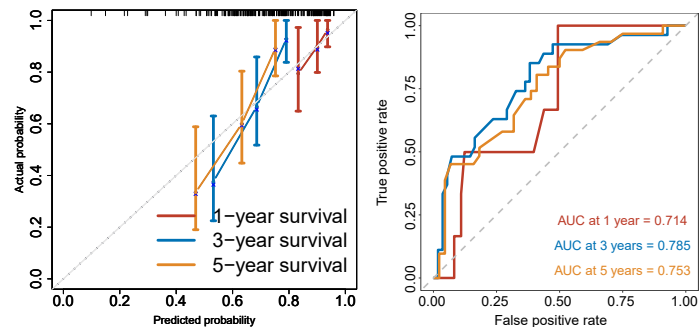

E

GSE68465

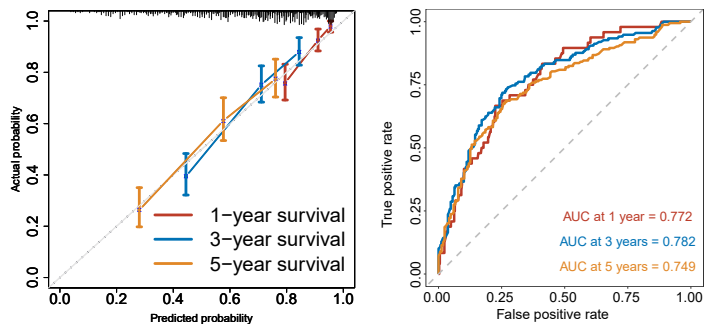

F

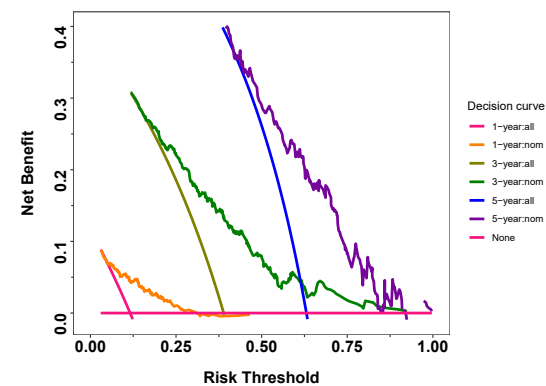

Supplement: Supplementary file 7 — Figure S7: jcmm70807‐sup‐0007‐FigureS7.pdf. [file JCMM-29-e70807-s001.pdf]

A

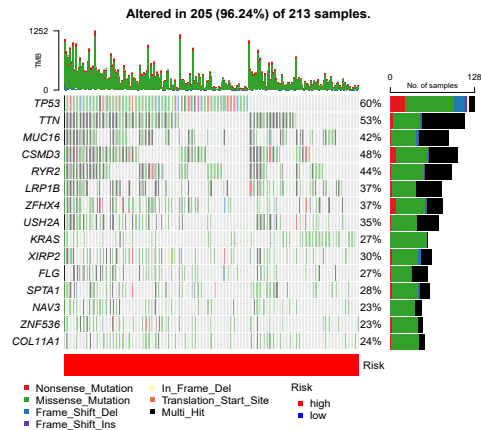

B

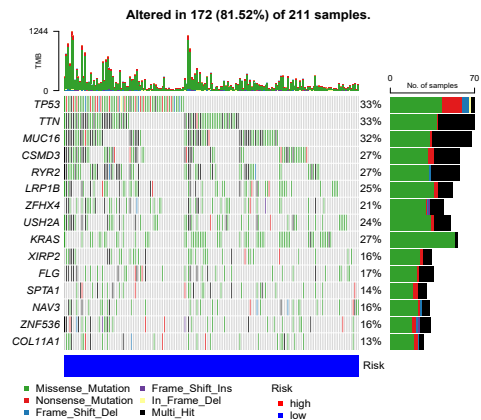

C

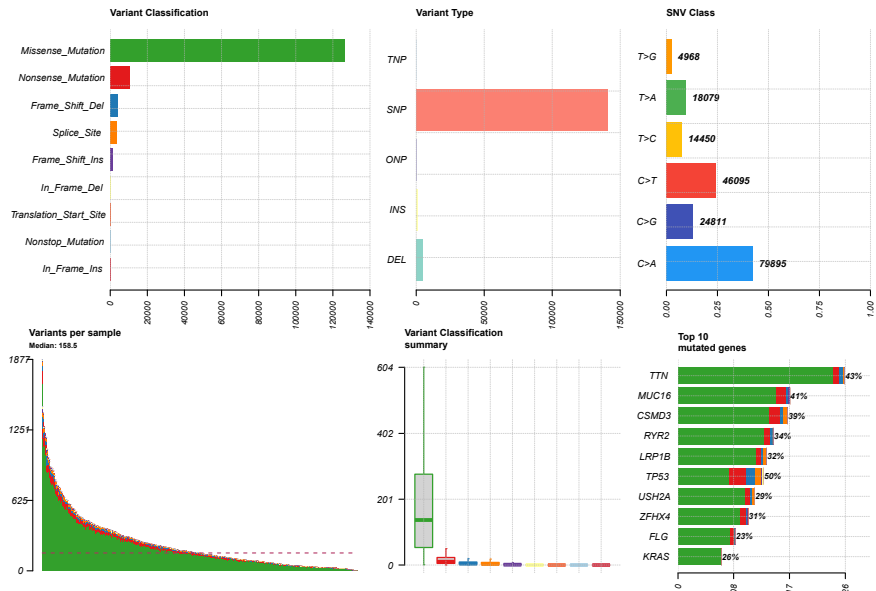

D

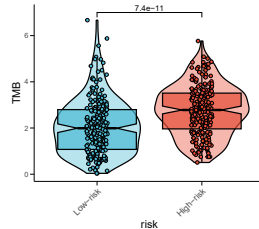

E

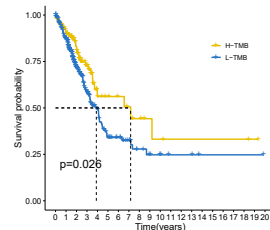

F

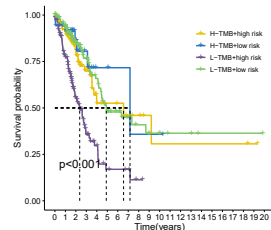

Supplement: Supplementary file 8 — Figure S8: jcmm70807‐sup‐0008‐FigureS8.pdf. [file JCMM-29-e70807-s004.pdf]

A

PLK1+ispinesib

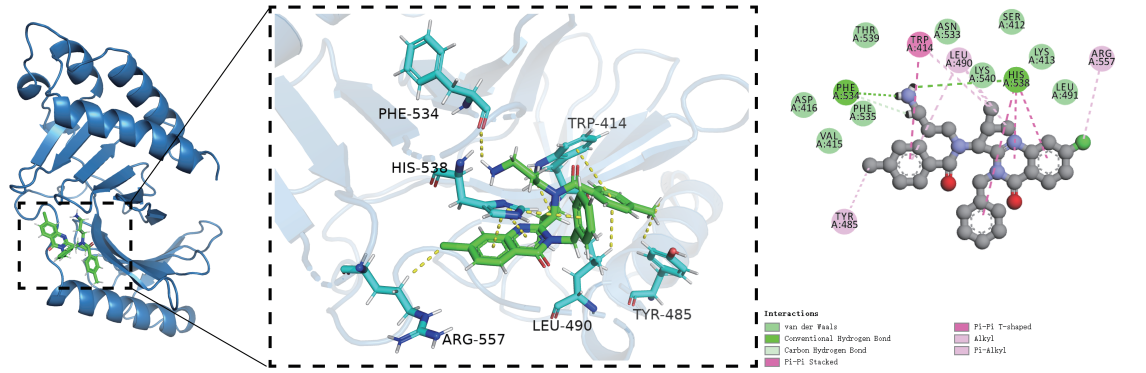

B

HMMR+SB-743921

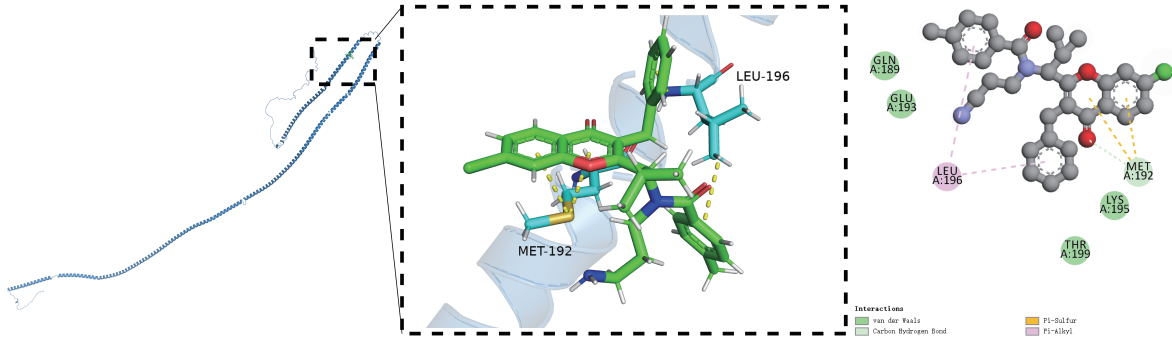

C

HMMR+ispinesib

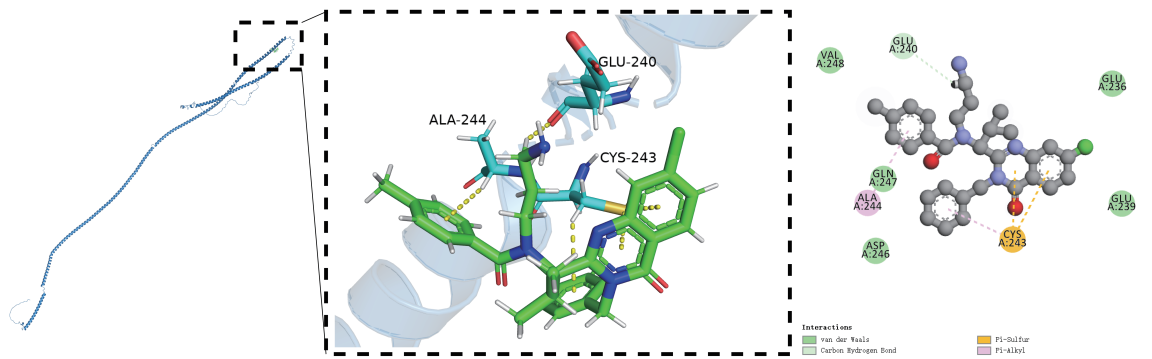

D

PRC1+SB-743921

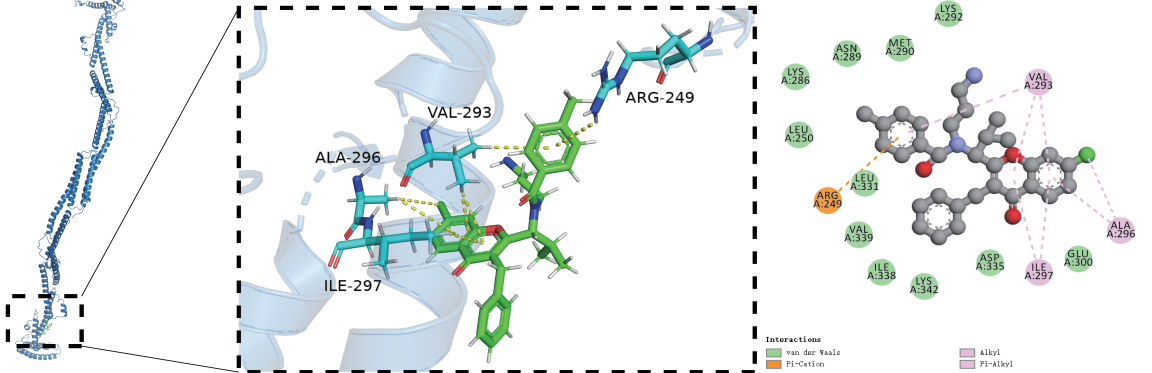

Supplement: Supplementary file 9 — Figure S9: jcmm70807‐sup‐0009‐FigureS9.pdf. [file JCMM-29-e70807-s013.pdf]

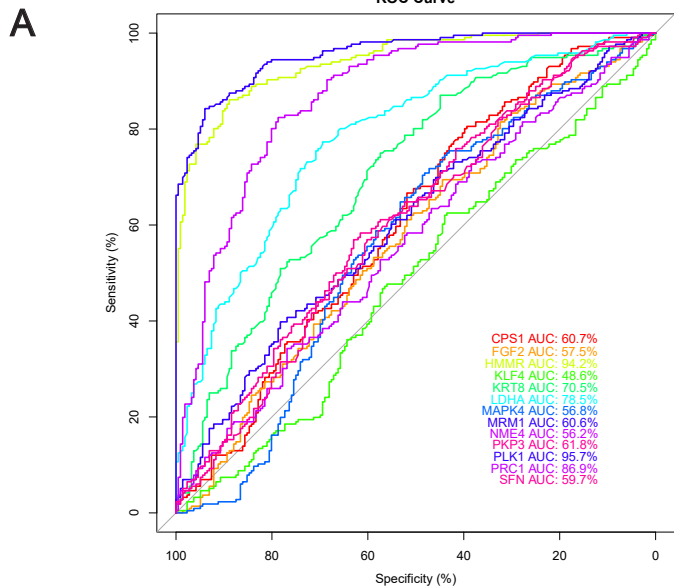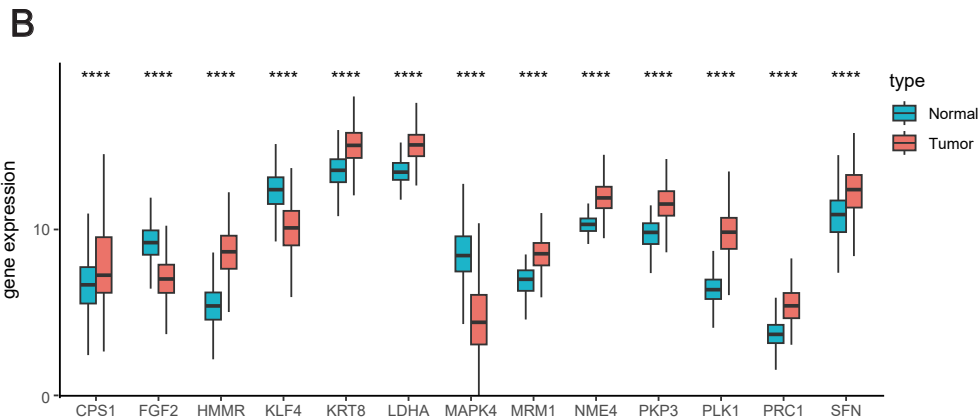

Supplement: Supplementary file 10 — Figure S10: jcmm70807‐sup‐0010‐FigureS10.pdf. [file JCMM-29-e70807-s002.pdf]

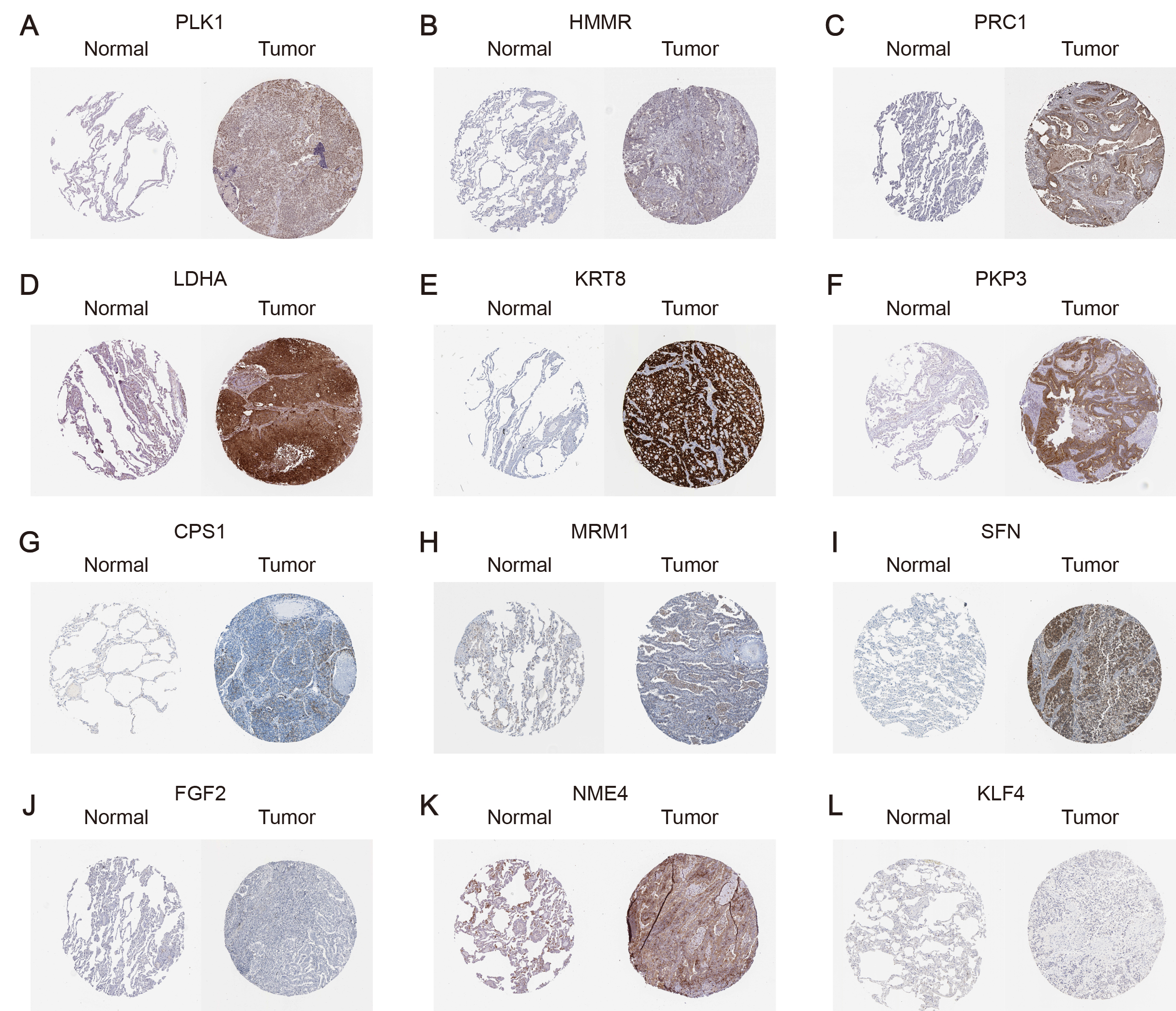

Supplement: Supplementary file 11 — Figure S11: jcmm70807‐sup‐0011‐FigureS11.jpg. [file JCMM-29-e70807-s005.jpg]

A

## HMMR

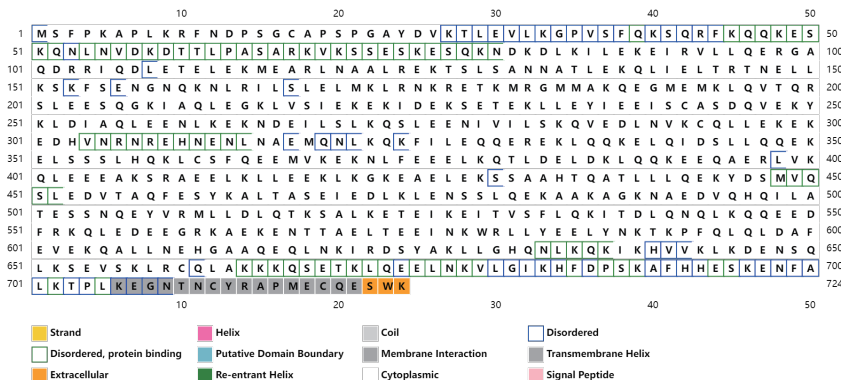

B

## PLK1

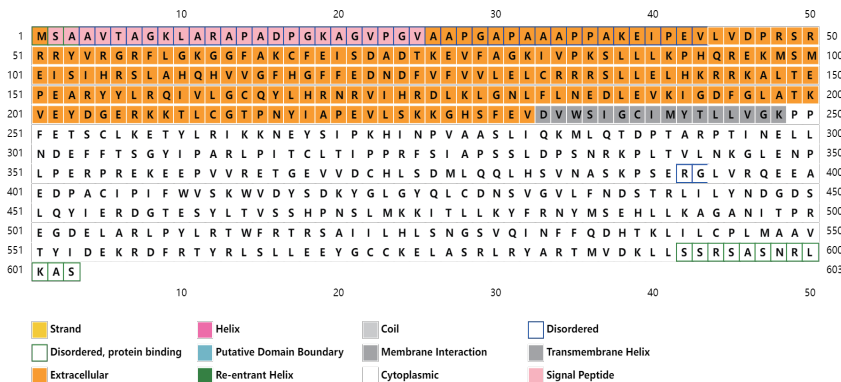

C

## PRC1

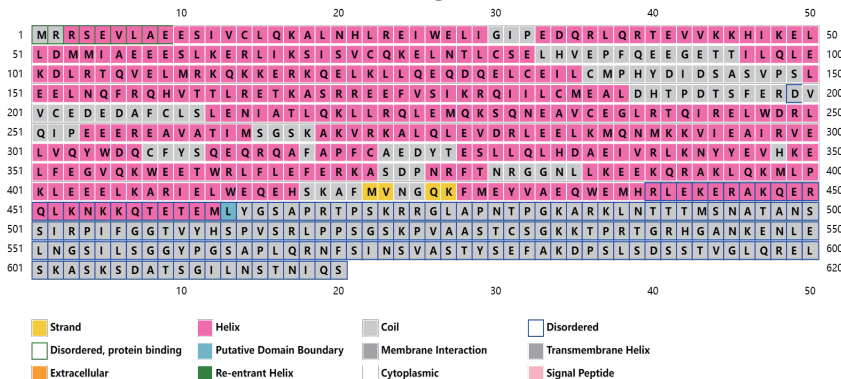

Supplement: Supplementary file 12 — Figure S12: jcmm70807‐sup‐0012‐FigureS12.pdf. [file JCMM-29-e70807-s011.pdf]
